# The Impact of a Ligand Binding on Strand Migration in the SAM-I Riboswitch

**DOI:** 10.1371/journal.pcbi.1003069

**Published:** 2013-05-16

**Authors:** Wei Huang, Joohyun Kim, Shantenu Jha, Fareed Aboul-ela

**Affiliations:** 1 Department of Biological Science, Louisiana State University, Baton Rouge, Louisiana, United States of America; 2 Center for Computation & Technology, Louisiana State University, Baton Rouge, Louisiana, United States of America; 3 Department of Electrical and Computer Engineering, Rutgers University, Piscataway, New Jersey, United States of America; University of Maryland, Baltimore, United States of America

## Abstract

Riboswitches sense cellular concentrations of small molecules and use this information to adjust synthesis rates of related metabolites. Riboswitches include an aptamer domain to detect the ligand and an expression platform to control gene expression. Previous structural studies of riboswitches largely focused on aptamers, truncating the expression domain to suppress conformational switching. To link ligand/aptamer binding to conformational switching, we constructed models of an S-adenosyl methionine (SAM)-I riboswitch RNA segment incorporating elements of the expression platform, allowing formation of an antiterminator (AT) helix. Using Anton, a computer specially developed for long timescale Molecular Dynamics (MD), we simulated an extended (three microseconds) MD trajectory with SAM bound to a modeled riboswitch RNA segment. Remarkably, we observed a strand migration, converting three base pairs from an antiterminator (AT) helix, characteristic of the transcription ON state, to a P1 helix, characteristic of the OFF state. This conformational switching towards the OFF state is observed only in the presence of SAM. Among seven extended trajectories with three starting structures, the presence of SAM enhances the trend towards the OFF state for two out of three starting structures tested. Our simulation provides a visual demonstration of how a small molecule (<500 MW) binding to a limited surface can trigger a large scale conformational rearrangement in a 40 kDa RNA by perturbing the Free Energy Landscape. Such a mechanism can explain minimal requirements for SAM binding and transcription termination for SAM-I riboswitches previously reported experimentally.

## Author Summary

Folding dynamics is crucial for RNA function. Riboswitches are a classic example. A typical riboswitch senses the cellular concentration of a small molecule. By refolding itself into a new structure, the riboswitch converts that information into changes in rates for synthesis of related metabolites. Understanding how the small molecule physically changes RNA structure can help us to target riboswitches, which occur mainly in bacteria, for drug design, or to engineer new riboswitches. This understanding has been blocked because 1) we cannot view intermediate stages experimentally and 2) simulations cannot reach the timescale for the structural conversion. Recent advances in RNA structure modeling enable us to model intermediate states. A new computer specialized for long timescale molecular dynamics (MD) simulations, called Anton, helps us to extend the simulation timescale. We modeled intermediate riboswitch structures, focusing on a reduced segment of the structure-switching region, in order to reduce the time required for a transition. We simulated an MD trajectory in which a small molecule converted the structure of this reduced switching region. Some steps in riboswitch structural transitions are therefore accessible to the newly extended MD timescale. Wider availability of resources like Anton can aid the advancement of riboswitch engineering and novel antibiotic design.

## Introduction

Riboswitches reveal the versatility of Ribonucleic Acid (RNA) folding, and its remarkable biological impact. They are folded mRNAs that sense cellular metabolite levels and control expression of downstream genes [1]–[4]. Design of altered or novel riboswitches has been suggested for bioengineering applications [5]–[9]. Riboswitches also represent an important target for the design of novel antibacterials [10]–[12].

**Figure 1:**
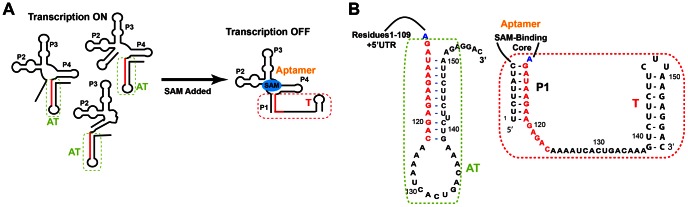
Schematic illustration of regulation of transcription by the SAM-I riboswitch in response to SAM. (A) At low levels of SAM (left), the riboswitch forms an ensemble of secondary structures, most of which include the formation of the antiterminator (AT) helix. As SAM levels increase, ligand binding induces the formation of the structure shown at the right. SAM is bound to the four helix junction in which the P1 helix prevents formation of the AT by sequestering the “switching strand” (highlighted in red). The formation of the rho-independent terminator hairpin (T) then terminates transcription. The region incorporating the AT/T formation is called the “expression platform” as it controls gene expression, while the four helix junction is termed the “aptamer”. (B) Closeup of the competing P1, AT, and T helices showing the explicit base sequences.

Riboswitches contain an aptamer, which recognizes and binds the metabolite. This binding triggers conformational rearrangement of the expression platform, which controls gene expression. Like other transcriptional riboswitches, the SAM-I riboswitch secondary structure is rearranged upon ligand binding [2], [13], [14]. The P1 and terminator (T) helices form in the ligand-bound state (Figure 1). This bound state is called the transcription OFF state since the terminator stops transcription. Without ligand the antiterminator (AT) helix forms, preventing formation of P1 and T helices, and allowing transcription (the transcription ON state).

SAM-I and other riboswitches raise the question–how can a small molecule binding to a limited contact surface cause a major folding rearrangement of a much larger RNA? Addressing this question requires consideration of conformational dynamics. X-ray studies of riboswitches have largely focused on the ligand-bound aptamer, truncating the expression domain to suppress conformational dynamics [15]–[18]. Such dynamic behavior is problematic for high resolution structure determination.

All-atom MD simulations are a major workhorse to tackle conformational dynamics [19]–[25]. Such methods have been applied to riboswitches, working largely with aptamer X-ray coordinates. These studies have revealed further insights into ligand recognition [26]–[30], the role of ions [25], [31], and contrasted dynamic properties in the liganded and unliganded states [27], [29], [30], [32], [33]. Larger-scale, slower dynamic processes have required coarse-grained modeling or directed simulations using biased force fields [34], [35].

Until recently, however, all-atom MD simulations using unbiased force fields have been generally limited to time scales less than microseconds. The birth of a specialized machine designed for MD simulation-Anton [36], [37] has increased the timescale limitation up to 200 times compared to simulations with conventional High Performance Computing (HPC) machines. Recent advances in software development and RNA structure modeling have improved the building of RNA models [38]–[48]. Together with enhanced sampling techniques [49]–[52] these modeling tools extend the accessible conformational space beyond that available within even the extended MD timescales.

Here we employ all-atom MD simulations to observe the direct effects of ligand binding on the equilibrium between alternative SAM-I riboswitch base pairing configurations. A large gap remains between the timescale required for strand migration (perhaps ms-seconds [53], [54]) and even the extended Anton MD timescale. We bridge this gap by bypassing the “nucleation” step in strand migration to simulate propagation-presumably a more rapid step. To generate “pre-nucleated” starting models for intermediate states, we capitalize on the recent advances mentioned above for sampling of RNA conformations.

We focus on the relationship between SAM binding and P1 helix propagation, or strand migration from an AT to a P1 helix (also termed the “switching” event [29] or “conformational collapse” [55]). We observed a strand migration event in the presence of SAM converting 3 AT helix base pairs (characteristic of the unbound riboswitch ON state) to competing P1 helix base pairs (characteristic of the OFF state). Overall, our simulations predict that SAM perturbs the reduced Free Energy Landscape (FEL) in a manner that favors conformations with expanded P1 helix base pairing and reduced AT pairing within the competing region, for certain starting geometries. Based on this simulation, we propose a mechanism for ligand-induced conformational switching which is consistent with reported requirements for SAM-I riboswitch function.

## Results

### Generation and structural analysis of models from MC-Sym

**Figure 2:**
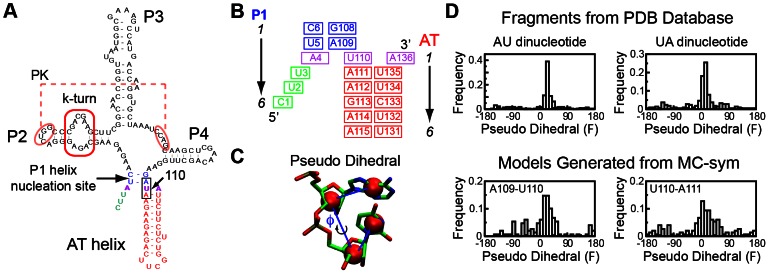
Structure modeling, and pseudo-dihedral analysis. (A) A secondary structure representation of the RNA switching intermediate which we chose to model for our starting structure is shown. The AT helix is assumed to be fully formed and two base pairs form in the P1 helix (in blue). The remaining P2, P3, and P4 helices and tertiary structures including a pseudoknot structure and a kink-turn motif (red box) are assumed to form and derived without change from the aptamer X-ray structure. The base triplet highlighted in purple: A4 (P1 helix, 5′ strand)-U110 (switching strand)-A136 (AT helix, 3′ strand) represents one base pairing position that involves the P1 helix and the AT helix competition. Three additional nucleotides (in green) in the 5′ strand of the P1 helix are modeled as single strand RNA. Thus, these three positions begin the simulation as AT base pairs. B). Schematic of the local switching region. The two arrows indicate the order for numbering the base pairs. C) Schematic of the pseudo-dihedral angle definition from reference [58] D) Histogram of pseudo-dihedral angles for 5′-AU-3′ dinucleotide (Left) and 5′-UA-3′ dinucleotide (Right) in known RNA structures. (Top) and for A109-U110 dinucleotide and U110-A111 dinucleotide sampled by MC-Sym (Bottom).

For SAM binding to fully convert an AT helix to a P1 helix may require at least milliseconds, judging from NMR measurements on an analogous strand-switching RNA [53], or longer based on a strand displacement assay [54]. We reasoned that the most rapid effect of SAM binding on the riboswitch would take place if the ligand bound to an intermediate conformation, hybridizing elements of the ON and the OFF state. Figure 2 shows a schematic of the strategy that we used to generate a starting configuration for our MD simulation. In the ON state, one strand of the P1 helix pairs with a downstream segment of the expression domain (removed in the crystallized RNAs) to form the AT helix. We initiated our simulation with a truncated segment fixing a partial P1 helix (two base pairs), and a partial AT helix (seven base pairs). In between a 4 nucleotide competition region can form either a P1 or AT helix. We call this “hybrid” construct 6P1_11AT, since it has the potential to form up to 6 P1 base pairs and up to 11 AT base pairs. Our simulations start with three of the four switching base pairs as AT helix, and a boundary nucleotide residue (U110) is positioned equally close to its putative AT or P1 binding partners.

This choice of starting structure allowed us to 1) Work with a segment that was shown experimentally to bind SAM, 2) Include the minimal nucleated P1 helix known to bind SAM, and 3) Maximize the potential number of AT base pairs with the potential to switch to P1 pairing (see “Details of MD simulations” in Supplementary Information (text S1)).

We used MC-Sym [40], [56] to sample the placement of the AT helix in the 3D structures and the geometry of the boundary region with the nucleated P1. Previously we showed experimentally that SAM binds to hybrid constructs [57]. Though reduced in affinity, the SAM binding to the hybrids has similar dependence on Mg^2+^, and similar sensitivity to mutations as with the aptamer. Therefore we assumed that the folding of the portion of the SAM/hybrid riboswitch complex outside of the strand switching region, henceforth referred to as the “aptamer core”, is similar to that in the X-ray structure of the aptamer domain.

Since the AT helix approximates a canonical A form geometry, the critical local region to be sampled is the three nucleotide segment A109, U110 and A111. These three nucleotides act as a hinge to bridge the partial P1 helix and the nearly complete AT helix. Additionally, an explicit triplet constraint was applied on the three nucleotides highlighted in purple in Figure 2A and 2B (A4, U110 and A136). Two adenosines compete for base pairing with a U (Figure 2B). The scripts used to generate the models can be found in the SI.

An overview of the outcome from MC-Sym sampling is shown in Figure 2 using the pseudo-dihedral angle [58]. Monitoring of the pseudo-dihedral angle (Figure 2C) indicates that MC-Sym has focused on the populated geometries according to the known structures, but also has sampled exhaustively the full range of geometries (Figure 2D). There is a region (between 80 and 170 degrees) that is rarely sampled due to steric clash with the P3 helix coordinates (Figure 2D). Therefore, the results demonstrate that MC-Sym can sample a wide range of the conformational space, while placing the AT helix without steric clashes.

### Selection of starting models for MD simulation

**Figure 3:**
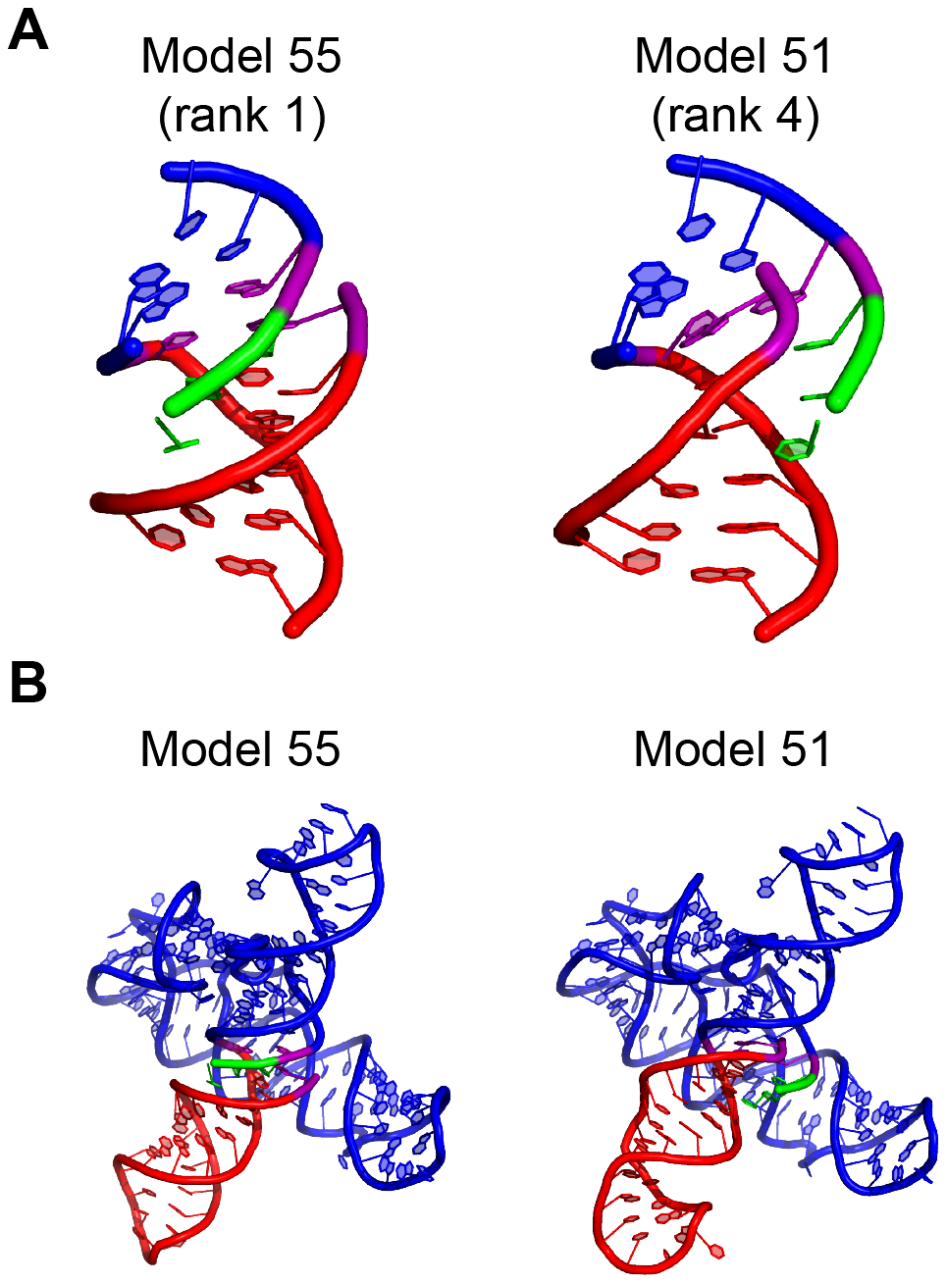
Models selected for MD simulations. (A) Closeup view of switching region for model 51 and model 55 in cartoon representation. (B) Global view of model 51 and model 55 in cartoon representation.

Three criteria were used for selecting MC-Sym generated models for MD simulations: 1) Calculated potential energy should be favorable, 2) The SAM binding pocket must be accessible and 3) Coaxial stacking should be present between the P1 and AT helices. The latter constraint was based on experimental observations that SAM binding at µM affinity was detected for RNA constructs which allow the potential for such stacking (“3P1_10AT”), but not for those which do not (“3P1_9AT”) [57]. For reasons explained in supplementary information, we used the Amber99bsc0 force field with the generalized Born (GB) implicit solvent model to calculate free energy. Two (model 51 and model 55) out of the top five ranked in terms of free energy satisfied all the three criteria. Figure S1A shows calculated free energies, while Figure S1B highlights the coaxial stacking for these two models as measured by internucleotide vdW energies. The local geometry of the switching region is displayed schematically for these two structures in Figure 3A and global folds are shown in Figure 3B. The main difference between these two models is that the unpaired 5′ strand of the P1 helix is placed in the two different grooves of the AT helix–in the minor groove of the AT helix for model 51, and in the major groove for model 55 (Figure 3A).

The geometry sampled in model 51 and 55 resembles an RNA triple helix composed of poly(U)-poly(A)-poly(U) from a crystal structure [59]. With limited experimental data, these two models are rationalized as potential models for the intermediate or “transition state” between ON and OFF state.

### Strand migration is observed in model 51 with SAM present

**Figure 4:**
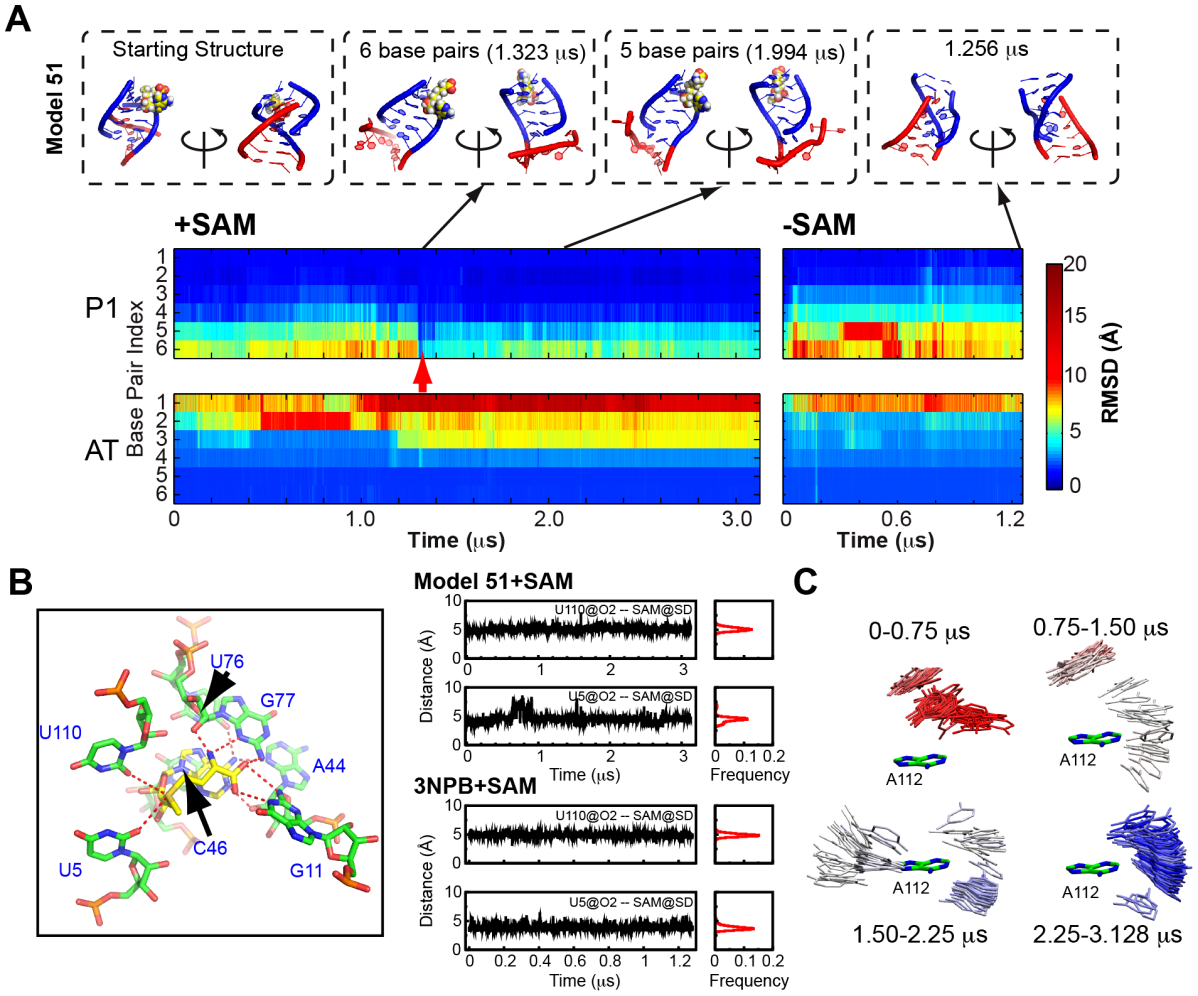
Monitor of strand switching event in model 51 simulations. (A) Time evolution of RMSD for individual base pairs in the P1 and the AT helix from simulations on model 51 in the presence and in the absence of SAM. The crystal structure of the P1 helix from *yitJ* was used as the reference structure for the P1 helix, while a standard A-form helix was used as reference for the AT helix. The starting structure and some snapshots from the trajectories are shown around the RMSD plot. The red arrow highlights the appearance of the complete P1 in model 51 with SAM. (B) RNA-ligand interaction distance monitor. (Left) Highlight of the interaction between SAM and nucleotides in the binding pocket observed in the crystal structure [60]. The adenine moiety of SAM stacks on nucleotide residue C46. The adenine ring and methionine moiety of SAM form a network of hydrogen bonds with nucleotides in J1/2 and P3 (G11, A44, G76 and G77). The readout of the positively charged sulfur on SAM is achieved by two carbonyl oxygen atoms (O2) on U5 and U110. The numbering of nucleotide residues follows that in 6P1_11AT. (Right) Monitor of the distances for the electrostatic interactions between RNA and SAM during the simulation for model 51 and 3NPB in the presence of SAM. (C) Relative positions of U2 to A112 during each 750 ns segment of the simulation for model 51 with SAM. U2 is shown for every 100 snapshot, the color scale is defined by the time step of the snapshot from red to blue.

Table 1 lists MD trajectories included in this study, using model 51 and 55 and the X-ray coordinates (3NPB) [60] as starting models. Different trajectory evolutions are observed for model 51 with or without SAM. Strikingly, formation of a complete P1 helix (all 6 Watson-Crick base pairs) is observed at ∼1.3 µs for the simulation in the presence of SAM (see Movie S1). Figure 4A displays the time evolution of RMSD for individual base pairs with reference to that in the P1 helix of the X-ray structure. A small RMSD value (deep blue) indicates that the geometry of the nucleobases in a single base pair is close to that observed for the Watson-Crick base pair in the crystal structure. Monitors of classical Watson-Crick hydrogen bonding presence for the base pairs in the P1 helix and the AT helix are presented in Figure S2.

**Table 1:** Summary of MD trajectories obtained using Anton.

Starting Coordinates	+/− SAM	Total trajectory Time (µs)	Description/summary of outcome
Model 51	+	3.1	Transient conversion to full (6 bp) P1 helix (1.3 µs) near frame 6615
Model 51 (frame 6615-6 P1 bps)	+	1.767	Full (6 bp) P1 helix maintained
Model 51 (frame 9974-5 P1 bps)	+	1.213	5 base pairs of P1 helix maintained
Model 51	−	1.256	Loss of 2 P1 helix pairs
Model 51 (frame 6615-6 P1 bps)	−	3.076	Full P1 helix maintained
Model 51 (frame 9974-5 P1 bps)	−	1.927	∼1 base pair flips from P1 to AT helix
Model 55	+	1.467	Local minima with non-WC pairing
Model 55	−	0.642	Little change from starting base pairing
3NPB	+	1.28	Sustained P1 helix pairing
3NPB	−	0.87	Some P1 helix fraying

As is apparent from Figure 4A and Figure S2, the time at which the P1 helix was completely formed can be located as indicated with the red arrow in Figure 4A. The lifetime of this conformation spans from frame 6544 to frame 6635 (18.2 ns). The top 4 base pairs in the P1 helix (base pair 1 to 4) maintain the P1-like conformation corresponding to the crystal structure during the remaining simulation in the presence of SAM. Additionally, the electrostatic interactions between the sulfur atom of SAM and the carbonyl oxygen atoms of two U nucleotide residues persist through out the simulation (Figure 4B) as observed in the repeated simulation on the aptamer domain of the *yitJ* SAM-I riboswitch (3NPB in the presence of SAM in Table 1).

The short life span of the fully formed P1 helix is linked to fraying of the closing base pair (base pair 6). The two participating nucleotides flip to a cross-strand stacking conformation. The adjacent base pair (base pair 5) is disrupted shortly after the loss of the closing base pair, but reappears at 1.9 µs for 300 ns (altogether 2250 out of 9097 snapshots after the strand migration event display this base pair). The two bases remain proximal (Figure 4C), however, and flip between states involving alternative hydrogen bonding patterns (Movie S1, Figure S3).

A similar plot for the AT helix pairs shows that the destabilization of AT base pairs 1 to 3 precedes complete P1 formation (Figure 4A, Figure S2). The disruption of this AT region is not due to the deficiency in modeling the AT helix since the simulation on the same model without SAM maintains the geometry close to a standard A-form helix for 2 (base pair 2 and 3) out of these three base pairs. Moreover, the 2 base-pair starting partial P1 helix is unstable in the absence of SAM in model 51 (Figure 4A, Figure S2).

During the interval leading up to the strand migration event, P1 helix base pairs 4–6 show a slowly rising trend in RMSD relative to the X-ray coordinates (Figure 4A, Figure S3). Thus the strand migration event is preceded by a fluctuation in which the corresponding nucleotide residues explore a “transition state”. This fluctuation coincides with the loss of AT helix base pairs 2 and 3, which otherwise block P1 helix propagation through P1 base pairs 5 and 6. The RMSD for P1 helix base pairs 3–6 goes down, in some cases dramatically, at the time of the strand migration. The two terminal base pairs drift towards configurations which show only a slightly smaller RMSD relative to X-ray coordinates than at the start. The RMSD of backbone atoms relative to the X-ray coordinates, however, decreases and remains low after the strand migration event (Figure S3).

### Strand migration is dependent on starting geometry

Complete P1 formation does not take place in model 55 within the time scale (1.467 µs) accessible so far for this simulation when SAM is present. Base pair 3 in the P1 helix gets trapped in a state with base pair geometry close to an AU Hoogsteen base pair (U•A cis W.C./Hoogsteen and class XXIII according to reference [61]) (Figure S4). In addition, this state is stabilized by a new hydrogen bond interaction between A4 and SAM, which is not sampled during the simulation of the aptamer domain (the construct for the X-ray study) in the presence of SAM (Figure S4B, C).

### Analysis of trajectories in the presence and absence of SAM

**Figure 5:**
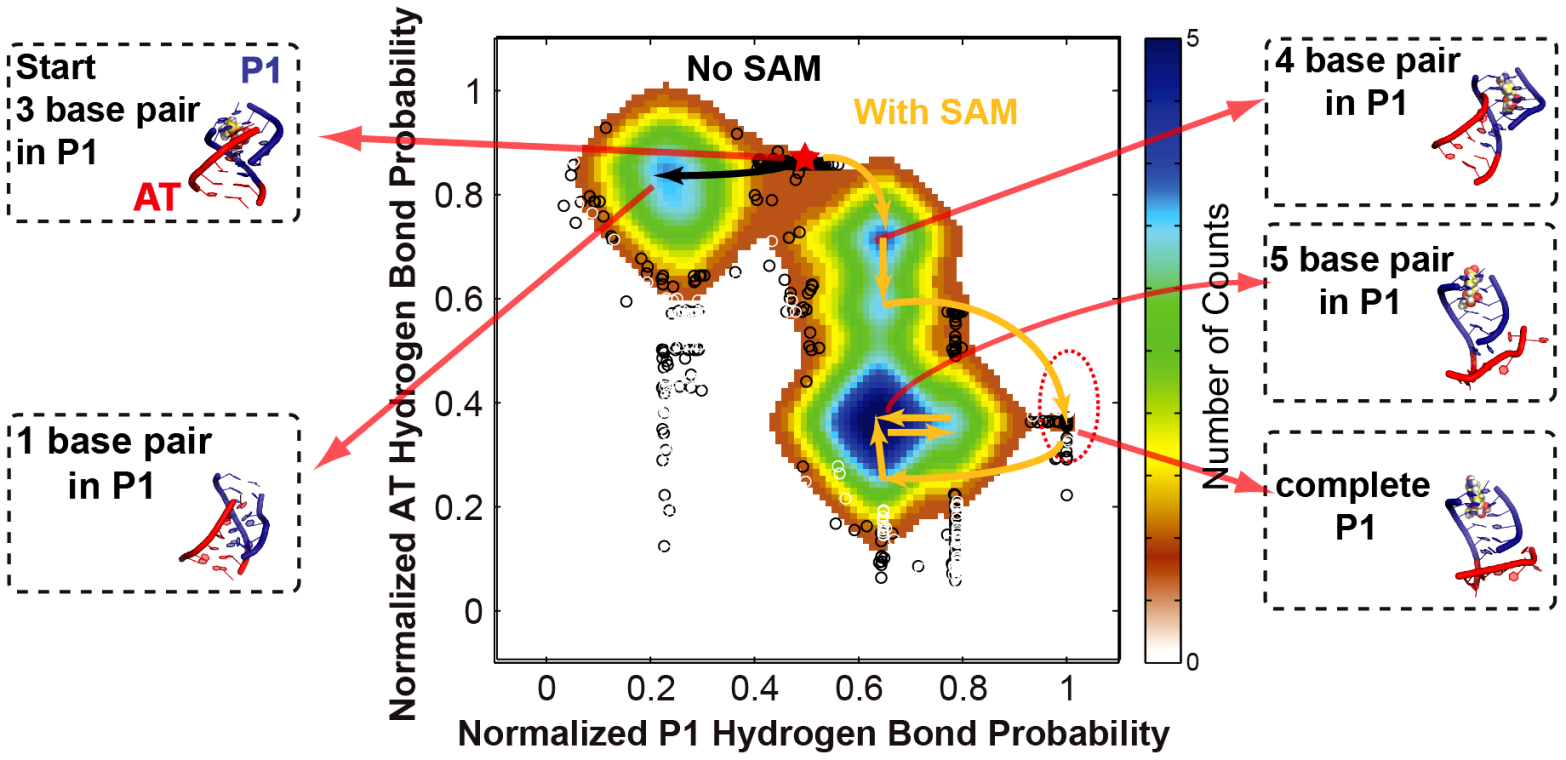
Visualization for simulations on model 51 over reduced conformational space representation. Two simulations include trajectory starting from model 51 in complex with SAM and trajectory starting from model 51 only. The red star indicates the position of the starting structure on the reduced conformational space. The black and orange arrows show some major transitions in temporal order for model 51 without SAM and with SAM respectively. The fractions of hydrogen bonds, represented by averaged hydrogen bond probability (see definition in Materials and Methods), in the P1 helix and in the AT helix are chosen as generalized reaction coordinates. The color scale is proportional to the logarithm of the population of snapshots on the corresponding reaction coordinates. The local switching regions from some representative snapshots are shown in cartoon representation with P1 in blue and AT in red. SAM is displayed in sphere model.

**Figure 6:**
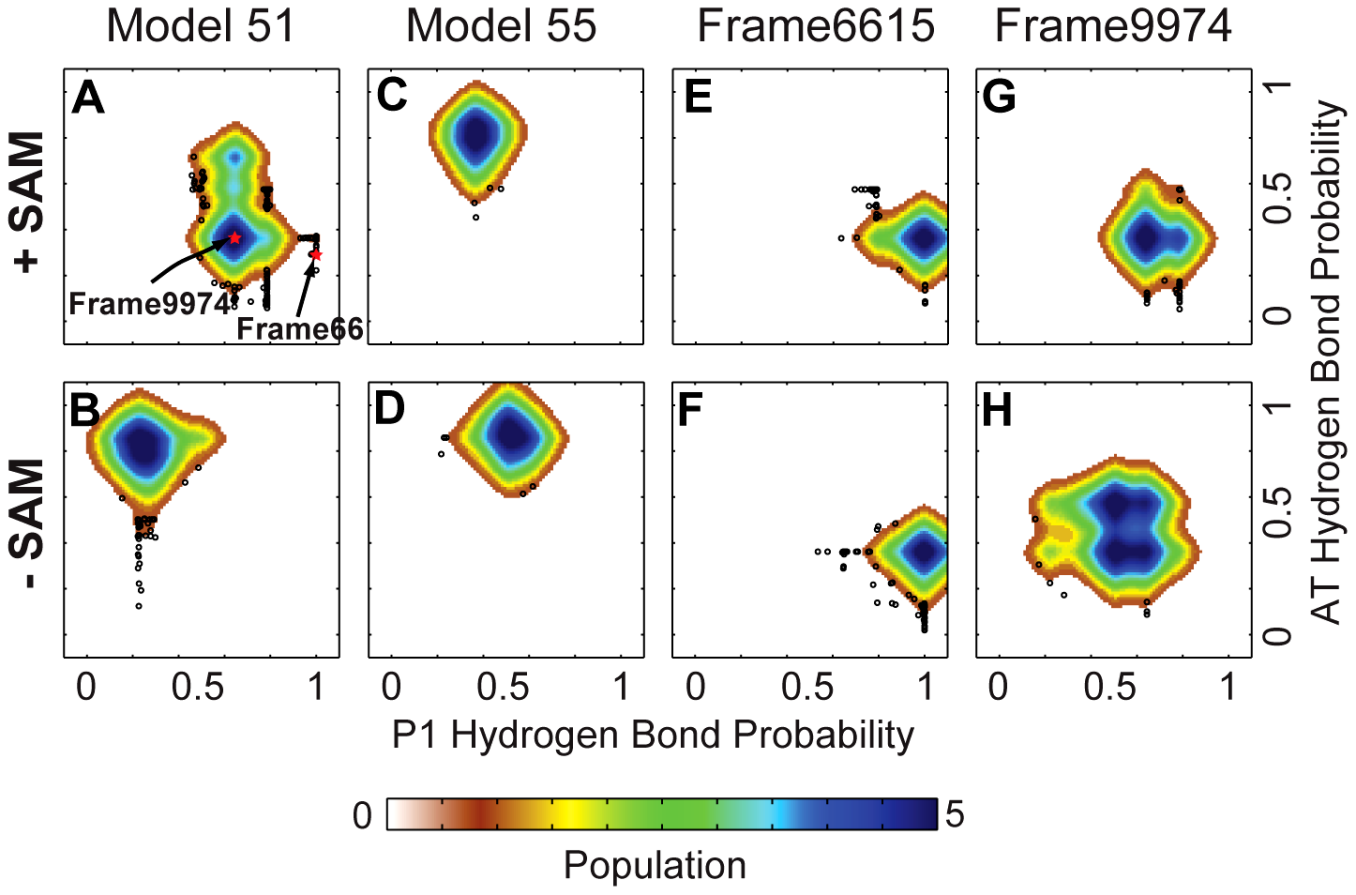
Visualization for simulations over reduced conformational space representation for individual model 51 and model 55 trajectories. The original trajectories (Figures 3 and 5) are shown here on the reduced conformational space separately (top) with (left) and without (right) SAM (A–B). The trajectories with or without SAM for model 55 are shown in a similar representation immediately below (C, D). Trajectories were initiated from frame 6615 (E, F) of the original model 51 trajectory with SAM at which a full 6 base pair P1 helix was observed, or frame 9974 (G, H), which contained 5 of 6 possible P1 helix pairs. The full 6 base pair P1 helix as formed during the original simulation following the strand migration event at 1.3 microseconds proved stable over the course of simulations with and without SAM (E, F, respectively). Simulations starting with frame 9974 displayed evidence of conversion of at least one base pair from P1 to AT helix pairing without SAM (H), whereas at least 4 P1 helix base pairs persisted throughout the simulation in the presence of SAM (G). Lengths of the trajectories are summarized in Table 1.

In Figures 5 and 6 we visualize the conformational pathways observed for the various trajectories for model 51. The overall fraction of hydrogen bonds in Watson-Crick base pairs from the P1 helix and from the AT helix are used as generalized coordinates. Figure 5 displays the conformational trajectories for a simulation started from model 51 only, and a second simulation from model 51 in complex with SAM.

The results suggest that starting model 51 locates at a branch point in the FEL. The formation of a stable AT helix (high probability for AT Helix Hydrogen Bonding-vertical axis) is favored in the absence of SAM, while the presence of SAM allows model 51 to navigate to other transient states and eventually leads to sampling of the conformation with a complete P1 (high probability of P1 helix formation-horizontal axis, Figure 5). However, the event of complete P1 helix formation is short-lived (Figure 5, Figure 6A, B). Therefore, a third simulation restarted from a snapshot with complete P1 at frame 6615 of the first trajectory in the presence of SAM (the snapshot with the lowest RMSD relative to the X-ray coordinates) was performed to evaluate the stability of this conformation (Figure 6 C, D). A 1.767 µs trajectory starting from frame 6615 in simulation of model 51 with SAM, only samples the bottom part of a deep energy funnel populated by an ensemble with complete P1 helix (Table 1, Figure 6E).

Interestingly, P1 helix base pairing is also relatively stable (persists through the simulation) when frame 6615 with a complete P1 helix is used as the starting coordinates for a simulation without SAM present (Table 1, Figure 6F). When snapshot 9974, with a five base pair P1 helix, is used as starting coordinates, the 5 P1 helix base pairs again remain relatively stable over the course of the trajectory (Table 1, Figure 6G). In this case, however, opening of some individual P1 helix base pairs is observed, particularly towards the end of the trajectory with SAM absent (Table 1, Figure 6H). In the latter trajectory, at least one P1 helix base pair reverts to AT base pairing.

### The role of SAM in pre-positioning J1/2 for P1 helix stabilization

**Figure 7:**
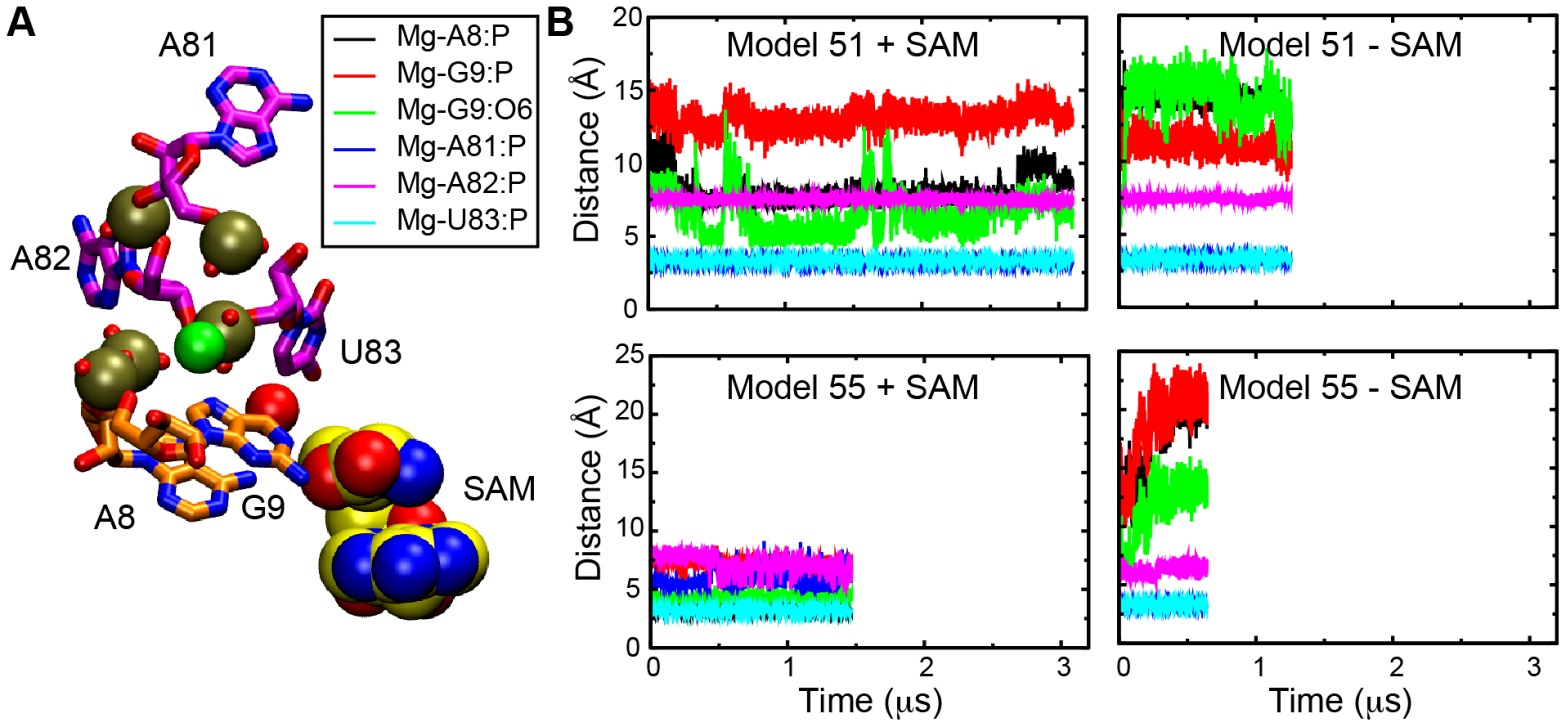
Monitor of Mg^2+^ bridging interaction with SAM, and nucleotides on J1/2 and J3/4. A) Geometry of Mg^2+^ binding site in SAM-binding region, involving coordination with electronegative groups on SAM, J1/2 and J3/4. B) Mg^2+^ contact distances with the electronegative groups listed in the legend four trajectories with and without SAM using model 51 and model 55 as starting coordinates. Contacts with phosphates on nucleotides 81–83 in J3/4 are persistent in the presence or absence of SAM. Contacts with two of the electronegative groups on J1/2 (A8 P and G9 O6) become short and persistent when SAM is present only.

In SAM-I aptamer X-ray structures a Mg^2+^ ion observed near the SAM binding site and phosphate moieties in J1/2 and J3/4 [60], [62]–[64]. We monitored the contact distances between this Mg^+2^ and phosphates in J1/2 in the various trajectories of the SAM-I riboswitch aptamer and hybrid starting models with and without SAM. Our previous simulation on another aptamer of SAM-I riboswitch—*metF* from *T.tengcongenesis* [31] indicated cooperativity between this Mg^2+^-phosphate coordination complex and SAM-leading to stabilization of tertiary interactions. Similarly, this effect was also observed in simulations of model 51 and 55 in the presence of SAM (Figure 7). The presence of SAM is correlated with the maintenance of short magnesium contacts with J1/2, while these contact distances increase during the simulations without SAM. Contact distances between Mg^2+^ and phosphates in J3/4 are almost constant in the presence and absence of SAM. For the *yitJ* aptamer the correlation between the presence of SAM and short Mg^2+^ contact distances with J1/2 is still maintained (Figure S5, but contact distances with phosphates on J3/4 begin to increase in the absence of SAM. Additionally, for restarted simulations of frame 6615 and 9974, the contacts of this Mg^2+^ ion with J1/2 are still maintained even in the absence of SAM (Figure S6). Overall, these results confirm the stable coordination between the Mg^2+^ ion and J3/4 in the absence of SAM, and the tendency of SAM contact to stabilize an additional coordination with J1/2.

Movies S2, S3, S4, S5, S6, S7 also highlight base moieties attached to nucleotide A7/9 in J1/2 and A80/82 in J3/4. Our earlier study also observed transient formation of a non-adjacent dinucleotide stack between nucleotide bases in J1/2 and J3/4 in simulations with and without SAM [31]. Of the ∼12 X-ray SAM-I riboswitch coordinate sets [60], [62]–[64] all except one (pdb id 3GX3, with SAH bound) show the two nucleotide bases pointing to the same region outside the helix, with the respective bases within 3–7 angstroms proximity. In this study we again observed transient formation of dinucleotide stacking with and without SAM for model 51 and the aptamer, but with alternating stacking geometries (Movies S2, S3, S4, S5, S6, S7). Predominantly the two nucleotides were positioned with favorable stacking energies (Figure S7) but little effect was observed from SAM binding.

## Discussion

### A mechanism for ligand-induced conformational switching

Overall, we can summarize the results with model 51 MD trajectories as the following: 1) In the absence of SAM, 2–3 starting P1 helix base pairs appear to be unstable, whereas a long AT helix remains stable up to the terminal base pair; 2) In the presence of SAM, a strand migration event is observed after ∼1.3 µs leading to transient formation of a full 6 base pair P1 helix, at the expense of competing AT helix base pairs; 3) Terminal base pairs within the fully formed P1 helix form transiently in the original simulation, but appear relatively stable in a new trajectory using the snapshot with fully-formed P1 helix as the starting point; 4) The fully formed P1 helix is also relatively stable in a trajectory which starts with the same snapshot even in the absence of SAM. 5) In a trajectory starting with 5 P1 base pairs with SAM, hydrogen bond contacts corresponding to the five base pairs appear slightly more stable than they do in one starting from the same snapshot without SAM.

By contrast, trajectories starting with model 55 result in a conformation in which the competing base pair at the boundary between P1 and AT base pairs forms a non-Watson-Crick pair, while other P1 and AT base pairs are stable. Taken together, these findings indicate that SAM binding promotes P1 helix base pairing at the expense of AT helix pairing, but with qualifications. Certain starting geometries, such as that in which the 5′ nucleotides reside near the major groove of the AT helix (as in model 55), may be slow to convert to the P1 helix-forming conformation. Our original simulation of model 51 in the presence of SAM resulted in 4 stable P1 helix base pairs. Thus, SAM binding may have its strongest direct stabilization of P1 helix base pairs near the SAM binding site.

All of these results are consistent with experimental evidence. A minimum length of P1 helix is necessary for SAM binding [13], [65], though the presence of a partial AT helix can restore µM SAM binding with a P1 helix as short as 2 base pairs [57]. The latter study indicated that SAM binding affinity increases in model systems as the P1 helix is extended and the AT helix shortened. There are also indications that P1 helix dynamics are reduced by SAM binding [17], [54], [65] for truncated aptamers.

### Mechanism for stabilization of P1 helix base pairing by SAM

Earlier we proposed that SAM contacts with J1/2 and indirect stabilization of Mg^2+^ contacts with J1/2 enhance P1 helix formation [31], and that the contacts with J1/2 block formation of competing conformers [66]. Our simulation suggests that additional enhancement of P1 helix formation arises through direct contact with SAM. The importance of these electrostatic SAM-P1 helix contacts for mediating the ligand binding specificity has been established experimentally [63].

As observed in our earlier simulations [31], direct contacts between SAM and the key G11 nucleotide within the P1 helix are persistent throughout these extended timescale simulations. In addition, we observed shorter contact distances between a bound Mg^2+^ and at least two electronegative functional groups on J1/2 in the presence of SAM during the simulations starting with model 51 and with the aptamer, than in the absence of SAM (Figures S4, S5, S6). The Mg^2+^ ion site which we have monitored here, observed in the original X-ray structures, is suspected to form an inner sphere coordination complex [31], [67].

Movies shown in supplementary materials (Movies S2, S3, S4, S5, S6, S7) vividly illustrate the interplay between SAM, Mg^2+^, and the backbones of the J1/2 and J3/4 junctions. Movies without SAM show the Mg^2+^ surrounded by phosphates from J3/4, with particularly stable coordination with phosphates 81 and 83 (83 and 85 in the aptamer). In the presence of SAM, G9/11 O6 is anchored in a bridging position between the Mg^2+^ and phosphate groups in J1/2. A recent study identified a cooperative effect between Mg^2+^ and SAM in SAM-I riboswitch folding, and proposed a role for the same core Mg^2+^ in pre-organizing folding intermediates for SAM binding [55]. Movies S2, S3, S4, S5, S6, S7 provide a striking illustration of a potential mechanism to explain this cooperativity. This coordination complex could induce a reorientation of the P1 helix, as reported [65], by fixing the position of J1/2.

Favorable non-adjacent dinucleotide stacking between nucleotide bases in J1/2 and J3/4 is observed in model 51 with SAM, but with altered geometry in the absence of SAM (Movies S2, S3). Altogether, these observations leave an open question as to the role that non-adjacent dinucleotide stacking may play in pre-positioning J1/2 and J3/4 in a manner that is favorable to aptamer formation and P1 helix formation specifically.

### Implications of restarted MD simulations

The simulation of model 51 in this study shows that the stabilization of the partial P1 helix by SAM anchors the 5′ strand of the P1 helix in an orientation that enables this single strand region to compete over the AT helix. This model is reminiscent of an NMR study on a small RNA system showing that the stabilization of a pre-formed helical region by a tetra loop increases the rate of conversion between two different hairpin folds [53]. In the riboswitch, SAM stabilization of the nucleated P1 helix may play a similar role.

Simulations on the same starting coordinates in the absence of SAM displayed the loss of all P1 helix base pairing. Conformations with three or fewer base pairs in the P1 helix may not be stable enough to prevent the formation of the AT helix in the absence of SAM. When a snapshot with fully formed P1 helix (frame 6615) was used as the starting structure an MD trajectory displayed a relatively stable P1 helix even in the absence of SAM. When frame 9974 with 5 P1 helix base pairs was used as the starting structure, all 5 P1 helix pairs remained stable in the presence of SAM. With these starting coordinates, however, as the simulation time approached 1 µs, the P1 helix began to show some instability in the absence of SAM. Therefore, differing degrees of shift of the conformational equilibrium amongst a series of conformational intermediates toward the OFF state with SAM facilitate the SAM-I riboswitch function as a dimmer switch. The most dramatic SAM binding effect is on a hybrid conformer with minimal P1 helix base pairing.

### Implications for SAM-I riboswitch folding in active transcription complexes

In the biological context, it is proposed that SAM binding takes place soon after the transcription of the aptamer-forming segment [68]. The conformation is then locked before full transcription of the antiterminator, a mechanism similar to that indicated for other transcriptional riboswitches [69], [70]. Such a mechanism is highly sensitive to the concentrations of reaction components-a recent report indicated that nucleotide levels dramatically alter the degree of kinetic control of a lysine riboswitch within active transcription complexes [71]. For the *yitJ* SAM-I riboswitch, moreover, partial AT helix formation can take place in the non-overlapping region, even in the presence of a full P1 helix. Our simulations indicate that the presence of SAM would prevent strand invasion by this partial AT and dissociation of the P1 helix in this scenario.

**Figure 8:**
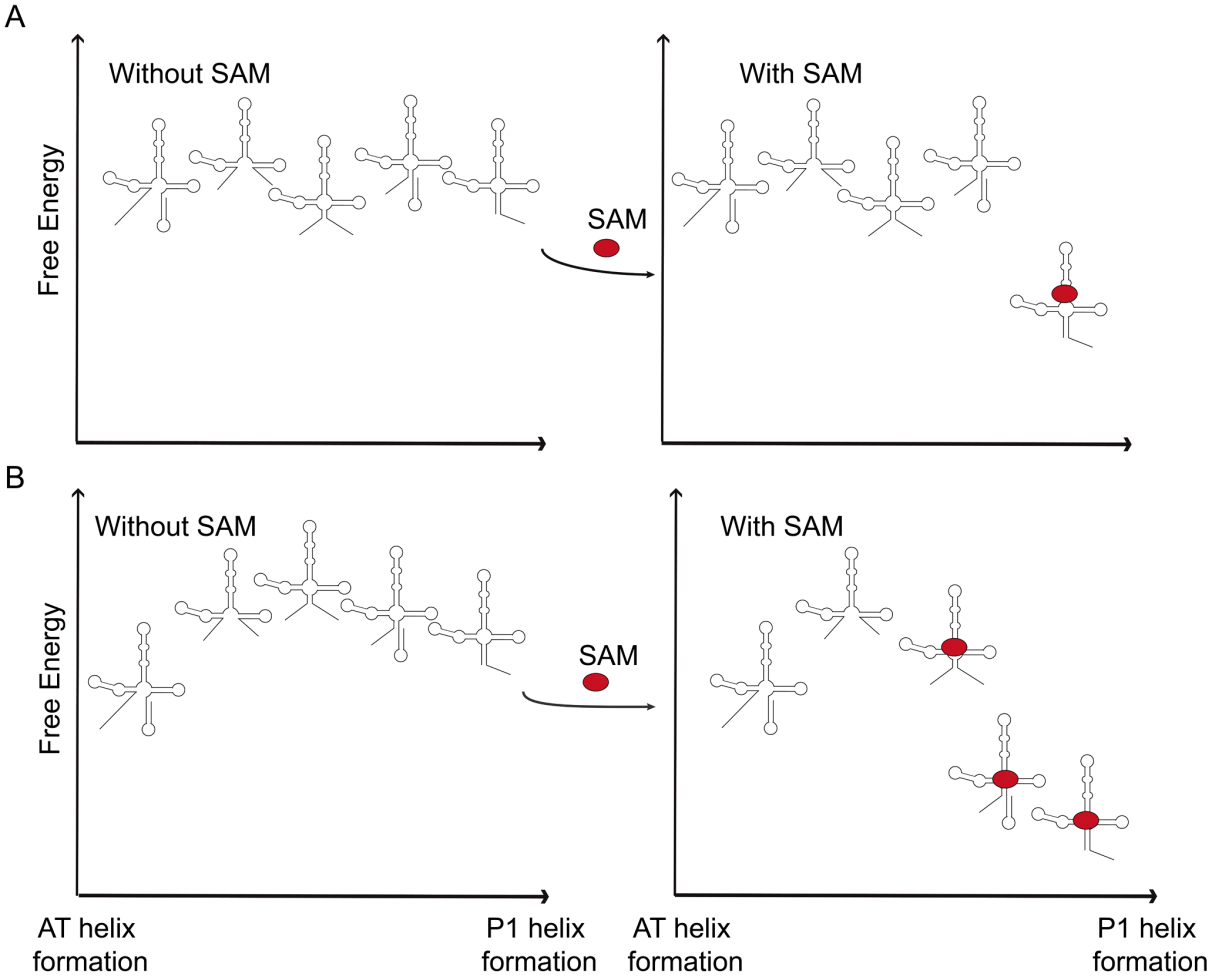
Schematic of conformational capture and FEL perturbation mechanisms for ligand-induced riboswitch conformational change. Each cartoon representation of the RNA molecule should be viewed as representing an ensemble of secondary structures with closely related topology in the P1/AT helix competition region. Conventional descriptions of “conformational capture” (A) assume sampling of numerous conformations, one of which is “selected” and fixed by ligand binding. If a subset of conformations is susceptible to reduced affinity ligand binding (B), SAM binding can lower the free energy of the partial P1 helix-forming families of conformers (third and fourth from the left in each panel). Note that in this situation, the ligand/aptamer complex can form rapidly through a pathway bypassing the non-P1 helix forming families of conformations (the two on the far left). Such a mechanism may be hypothesized for SAM-induced P1 helix formation *in vitro* or even during active transcription.

High resolution structures of riboswitch aptamers with and without ligand have led to the proposal that many fold according to the “conformational capture” mechanism [72], [73]. Typically this mechanism is described as selection by the ligand of a single bound conformer amongst a range of conformations being sampled by the unliganded substrate (Figure 8A). Inclusion of a portion of the expression domain, however, leads to more dramatic effects of ligand on RNA folding. Secondary structure calculations predict that an equilibrium Boltzmann ensemble for the *yitJ* SAM-I riboswitch includes some hybrid conformations with partial P1 and partial AT helix [57], [66]. A “capture” of these intermediates, according to simulations here, would facilitate rapid propagation of a longer P1 helix. This event would free a sufficient segment of the 3′ strand of the AT to nucleate the downstream Terminator sequence. By contrast, our simulations indicate that in the absence of SAM the AT helix could displace the nucleated P1 helix within the intermediates.

A more precise description of conformational capture in this scenario would be selection of a region of conformational space by the ligand, which then chaperones the RNA towards the aptamer configuration (Figure 8B). In panel B of the figure, free energy is now the relative free energy of the total system, including ligand as well as RNA plus solvent and ions. Conformations that can bind SAM have reduced free energy relative to those for which RNA and SAM are not in contact, and the reduction in free energy for each conformer is proportional to favorable free energy of binding. We hypothesize that the aptamer folding rate would be accelerated by this mechanism because during the Levinthal sampling process the FEL region that can initiate aptamer formation is widened.

Simulations of RNA folding kinetics [74] concluded that a strand migration pathway would lead to the fastest transition rate for an inter-conversion between two hairpins. Kinetic folding studies for a number of riboswitches [75]–[79] indicate that P1 helix formation takes place during later stages of the folding pathway. Our simulations and the experimental findings in our previous study [57] therefore raise the possibility of a role for SAM in accelerating P1 helix formation, by facilitating strand migration as the aptamer folding pathway. In this scenario, SAM binding could still facilitate aptamer formation after a portion of the expression domain has been transcribed. In vitro kinetics of SAM-I riboswitch folding and transcription termination would then be highly sensitive to mutations in the expression domain, as has been reported for a transcriptional lysine riboswitch [71]. SAM effects on P1 vs. AT length could be tested through NMR measurements on partially labeled SAM-I riboswitch hybrid constructs, or through NMR methods designed to detect minor conformers [80], [81].

### Approaching the switching event using MD simulations

In previous work we showed that altered base pairing in the unliganded *yitJ* SAM-I riboswitch as compared to the bound state extends beyond the P1/AT helix switch [57], [66]. The question of the impact of the ligand on the riboswitch conformation is therefore related to large-scale alterations in RNA folding. Although great advancement has been achieved to speedup MD simulations, a complete simulation of folding/unfolding for RNA of this size (∼40 kDa) is still not possible. The P1/AT helix switching event alone may take ms or longer, according to data on analogous model systems [53]. This is because the nucleation of the transient state takes up most of the folding time. The propagation step can be faster than the overall folding rate by four orders of magnitude [82]. Therefore, MC-Sym was used to sample a discrete conformational space aiming to identify candidate transient state models with atomic details to bypass the most time-consuming part of the simulation.

In the SI, we discuss measurements from the literature for folding and conformational switching for a range of RNAs. Overall, considering the literature data and estimating a rate constant based upon snapshots observed in the model 51 trajectory as transition states (Tables S1 and S2), the microsecond regime appears plausible for the strand migration within the three base pair stretch simulated in this study. Extension of the MD timescale to the microsecond regime by using Anton now appears to make some intermediate steps of strand migration accessible. The adequacy of force fields and parameters for long timescale simulations for RNA is relatively untested as compared to protein MD [83]. Nonetheless, it seems improbable that a strand exchange observed only in the presence of ligand, and leading to decreased RMSD relative to X-ray coordinates (Figure 4, Figure S3), is solely a result of instabilities or imperfections of the force fields [84]. The two terminal base pairs of the P1 helix are transient in the original model 51 simulation with SAM, although the RMSD relative to the X-ray structure for each base pair remains lower than before the strand invasion. This observation may reflect instabilities in the force field, a genuine tendency towards “fraying” [85], [86], or a longer simulation may be required to reach a thermodynamically stable state. The success of this study in observing a strand migration event should motivate efforts to optimize and validate parameters and protocols for long timescale MD simulations for RNA.

## Methods

### RNA modeling using MC-Sym

The atomic models for the RNA construct described in Figures 2 and 3 were generated using MC-Sym [40] installed locally. The aptamer core (highlighted in blue in the figure) was modeled using its counterpart in the known structure of the *yitJ* SAM-I riboswitch (PDB ID: 3NPB) [60]. The other parts of the construct were built from the library of small fragment RNA structures, known as Nucleotide Cyclic Motifs (NCMs) [56]. An explicit triplet constraint was applied on the three nucleotides highlighted in purple (A4, U110 and A136). In this way we sampled the 3D space in which these three nucleotides are proximal to each other. In these three nucleotides, the two As are competing for base pairing with a U. The scripts used to generate the models can be found in Appendix S2. Different RMSD threshold values were tested to ensure exhaustive sampling in the local region bridging the partial P1 and the AT helix (A109, U110 and A111). Models with small differences (low pairwise RMSD) in pseudo-dihedral angle of the A109-A111 region were filtered out. Energy minimizations (max step is 2000 or gradient tolerance <1.0) were performed on the atomic structures of the models generated from MC-Sym runs using Nucleic Acid Builder (NAB) [87]. AMBER99bsc0 force field [88] and Generalized Born model [89] with an inverse Debye-Huckel length of 0.19 Å^−1^ [90] were used in the energy minimization procedure. 149 models were generated in this step. This energy minimization is mainly to rebuild the chain connectivity for models generated from MC-Sym without introducing the sampling effect of the force field. Thus, we used MC-Sym to sample the possible placement of the AT helix in the 3D structures and the geometry of the potential nucleation site of the P1 helix close to the SAM binding pocket. The modeling assumed that the folding of the aptamer core is similar to that in the crystal structure of the aptamer domain. After the energy minimization step, models with high van der Waals energy were filtered out. There are two reasons for high van der Waals energy: 1) steric clashes that cannot be released by energy minimization, 2) broken chain connectivity that cannot be bridged during energy minimization.

### All-atom MD simulation

Models were chosen following the three criteria listed in the results section under “Selection of starting models for MD simulation”. For the models in the presence of SAM, the ligand was placed in the binding pocket while maintaining most of the interactions (except the contacts with the end base pair AU in the partial P1 helix) observed in the crystal structure of the aptamer domain complex (PDB: 3NPB). The simulations are run on Anton [36]. The equilibrated structures for Anton were prepared using local HPC clusters following the MD protocol as described in our previous study [31] (also see “Details of MD simulations” in Text S1). The trajectory was recorded for every 200 ps.

### Hydrogen bond probability

The definition of hydrogen bond probability (HBP) of the hydrogen bond i at time t is similar to that in reference [91]:
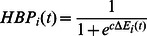
(1)where 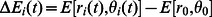, and 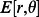 is defined as
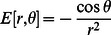
(2)Here 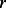 is the distance between hydrogen and hydrogen bond acceptor, 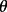 is the angle of hydrogen bond donor, hydrogen and hydrogen bond acceptor and scaling constant 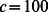 Å. In the reference state 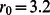 Å and 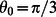 rad. The list of hydrogen bonds monitored is listed in Table S3.

## Supporting Information

Appendix S1 Predicting the time scale for the propagation step in strand switching.
(DOCX)

Appendix S2 MC-Sym scripts used to generate starting models for MD simulations.
(DOCX)

Figure S1 (A) Plot of the free energy rank with amber99 bsc0 and GB solvent model. (B) Scatter plot of vdW_A109-U110 versus vdW_U110-A111 clustered into 4 classes. Model 51 and Model 55 both fall within Cluster 2 (blue) indicating favourable VDW interactions through the A109-A11 junction region.
(TIF)

Figure S2 Hydrogen bond analysis of model 51 simulations. Monitor of hydrogen bonds in Watson-Crick base pairs. Here hydrogen bonds are defined with H-bond distance cutoff (<3.5 Å) and H-bond angle cutoff (>145°). The order of hydrogen bonds in the P1 helix is as in Table S3.
(TIF)

Figure S3 Clustering analysis for base pair 3–6 in the P1 helix for the model 51 trajectory with SAM. Each base pair was grouped into five states using *k*-means (*k* = 5) clustering based on the RMSD of nucleobases. (A) State assignments as a function of time for each base pair. The state assigned for each time point is indicated as an open circle. (B) Representative structure for each state. The centroid structure of each state was chosen to represent the state. The population of each state was labeled at the top of individual state. The RMSD for the base pair relative to X-ray coordinates is plotted against the vertical axis on the right. (C) The RMSD for the backbone for P1 helix base pairs 3–6 is plotted along with the same parameter for the trajectory of model 51 without SAM.
(TIF)

Figure S4 Monitor of some local geometries in model 55 simulations. (A) Time evolution of RMSD for individual base pairs in the P1 and the AT helix from simulations on model 55 in the presence (left) and the absence (right) of SAM, monitored as for model 51 in Figure 3. In contrast to the model 51 trajectories, no migration of P1 or AT helix base pairing is observed beyond the initially unpaired hinge position. (B) Local view of the switching region in model 55. The RNA is shown in cartoon representation except A4 and U100 in stick representation. The ligand SAM is also displayed in stick representation with carbon atoms in yellow. (C) Distance monitor during the simulations for model 55 and 3NPB with SAM for a AU Hoogsteen base pair (Top) and a new interaction between SAM and A4 sampled in model 55 (Middle). Monitor of the base stacking between A4 and A111 via vdW interaction energy (Bottom). The Hoogsteen base pairing scheme is shown on the right.
(TIF)

Figure S5 Plots of distance monitor for simulations of the *yitJ* aptamer (PDB ID: 3NPB) in the presence and the absence of SAM.
(TIF)

Figure S6 Plots of distance monitor for restarted simulations of model 51 for frame 6615 and frame 9974 in the presence and the absence of SAM.
(TIF)

Figure S7 (A) Monitor of vdW stacking energy calculated for non-adjacent dinucleotide stacking between nucleotides 7 and 82 for simulations of model 51 with (red) and without (black) SAM. (B) A histogram of calculated vdW values for the two nucleotides for each simulation.
(TIF)

Movie S1 Visualization of trajectory for model 51, with SAM, highlighting strand migration event converting 3 base pairs from AT to P1 helix configuration.
(MOV)

Movie S2 Visualization of SAM, core Mg^2+^, electronegative moieties in J1/2 and J3/4, and the dinucleotide stack between nucleotides A7/9 in J1/2 and A82/84 in J3/4. The bases in the non-adjacent dinucleotide stack are highlighted as sticks. G9/11 O6 is highlighted as a purple or red sphere. The Mg^2+^ ion is depicted as a yellow sphere. Backbone phosphates are also highlighted.
(M4V)

Movie S3 Similar visualization as for S2 for model 51 without SAM
(MPG)

Movie S4 Similar visualization for trajectory involving model 55 with SAM.
(MPG)

Movie S5 Similar visualization for trajectory involving model 55 without SAM.
(MPG)

Movie S6 Similar visualization for trajectory involving the aptamer, starting from x-ray coordinates, with SAM.
(M4V)

Movie S7 Similar visualization for trajectory involving the aptamer, starting from x-ray coordinates, without SAM.
(MPG)

Text S1 Supplementary Text includes descriptions of: Analysis of MC-sym sampling using the pseudo-dihedral angle, Ranking and selection of MC-sym modeled structures for MD simulations, Supplementary Methods (Details of MD simulations), Captions for supplementary figures and movies.
(DOCX)

Table S1 Calculated Free Energies (kcal/mol) using RNAeval (30) for formation of variant secondary structures of the riboswitch conformation shown in Figure 1
(DOCX)

Table S2 Calculated Free Energies using RNAeval for putative secondary structures and transition states from the study by Wenter et al. 2006.
(DOCX)

Table S3 List of hydrogen bonds included in calculations shown in Figures 5&6 and Figure S2.
(DOCX)

## References

[1] Roth A, Breaker RR (2009) The structural and functional diversity of metabolite-binding riboswitches. Annual Review of Biochemistry 78: 305–334. 10.1146/annurev.biochem.78.070507.135656 PMC5325118 19298181

[2] McDaniel BAM, Grundy FJ, Artsimovitch I, Henkin TM (2003) Transcription termination control of the S box system: Direct measurement of S-adenosylmethionine by the leader RNA. Proceedings of the National Academy of Sciences, USA 100: 3083–3088. 10.1073/pnas.0630422100 PMC152250 12626738

[3] Dambach MD, Winkler WC (2009) Expanding roles for metabolite-sensing regulatory RNAs. Current Opinion in Microbiology 12: 161–169. 19250859 10.1016/j.mib.2009.01.012 PMC2846827

[4] Haller A, Souliére MF, Micura R (2011) The Dynamic Nature of RNA as Key to Understanding Riboswitch Mechanisms. Accounts of Chemical Research 44: 1339–1348. 21678902 10.1021/ar200035g

[5] Muranaka N, Yokobayashi Y (2010) Posttranscriptional Signal Integration of Engineered Riboswitches Yields Band-Pass Output. Angewandte Chemie International Edition 49: 4653–4655. 20480477 10.1002/anie.201001482

[6] Topp S, Gallivan JP (2010) Emerging Applications of Riboswitches in Chemical Biology. ACS Chemical Biology 5: 139–148. 20050612 10.1021/cb900278x PMC2811087

[7] Dixon N, Duncan JN, Geerlings T, Dunstan MS, McCarthy JEG, et al (2010) Reengineering orthogonally selective riboswitches. Proceedings of the National Academy of Sciences, USA 107: 2830–2835. 10.1073/pnas.0911209107 PMC2840279 20133756

[8] Jin Y, Huang J-D (2011) Engineering a portable riboswitch-LacP hybrid device for two-way gene regulation. Nucleic Acids Research 39: e131. 21803790 10.1093/nar/gkr609 PMC3201887

[9] Weigand JE, Schmidtke SR, Will TJ, Duchardt-Ferner E, Hammann C, et al (2011) Mechanistic insights into an engineered riboswitch: a switching element which confers riboswitch activity. Nucleic Acids Research 39: 3363–3372. 21149263 10.1093/nar/gkq946 PMC3082870

[10] Deigan KE, Ferré-D'Amaré AR (2011) Riboswitches: Discovery of Drugs That Target Bacterial Gene-Regulatory RNAs. Accounts of Chemical Research 44: 1329–1338. 21615107 10.1021/ar200039b PMC3193592

[11] Kim JN, Blount KF, Puskarz I, Lim J, Link KH, et al (2009) Design and antimicrobial action of purine analogues that bind guanine riboswitches. ACS Chemical Biology 4: 915–927. 19739679 10.1021/cb900146k PMC4140397

[12] Maciagiewicz I, Zhou S, Bergmeier SC, Hines JV (2011) Structure-activity studies of RNA-binding oxazolidinone derivatives. Bioorganic & Medicinal Chemistry Letters 21: 4524–4527. 21733684 10.1016/j.bmcl.2011.05.130 PMC3466080

[13] Winkler WC, Nahvi A, Sudarsan N, Barrick JE, Breaker RR (2003) An mRNA structure that controls gene expression by binding S-adenosylmethionine. Nature Struct Biology 10: 701–707. 10.1038/nsb967 12910260

[14] Epshtein V, Mironov AS, Nudler E (2003) The riboswitch-mediated control of sulfur metabolism in bacteria. Proc Natl Acad Sci, USA 100: 5052–5056. 12702767 10.1073/pnas.0531307100 PMC154296

[15] Serganov A, Patel DJ (2012) Molecular recognition and function of riboswitches. Current opinion in structural biology 22: 279–286. 22579413 10.1016/j.sbi.2012.04.005 PMC3744878

[16] Batey RT (2011) Recognition of S-adenosylmethionine by riboswitches. Wiley Interdisciplinary Reviews: RNA 2: 299–311. 21957011 10.1002/wrna.63 PMC3618691

[17] Stoddard CD, Montange RK, Hennelly SP, Rambo RP, Sanbonmatsu KY, et al (2010) Free state conformational sampling of the SAM-I riboswitch aptamer domain. Structure 18: 787–797. 20637415 10.1016/j.str.2010.04.006 PMC2917978

[18] Baird NJ, Ferré-D'Amaré AR (2010) Idiosyncratically tuned switching behavior of riboswitch aptamer domains revealed by comparative small-angle X-ray scattering analysis. RNA 16: 598–609. 20106958 10.1261/rna.1852310 PMC2822924

[19] Siegel JB, Zanghellini A, Lovick HM, Kiss G, Lambert AR, et al (2010) Computational Design of an Enzyme Catalyst for a Stereoselective Bimolecular Diels-Alder Reaction. Science 329: 309–313. 20647463 10.1126/science.1190239 PMC3241958

[20] Karplus M (2010) Role of conformation transitions in adenylate kinase. Proceedings of the National Academy of Sciences, USA 107: E71. 10.1073/pnas.1002180107 PMC2867848 20424124

[21] Formoso E, Matxain JM, Lopez X, York DM (2010) Molecular Dynamics Simulation of Bovine Pancreatic Ribonuclease A-CpA and Transition State-like Complexes. The Journal of Physical Chemistry B 114: 7371–7382. 20455590 10.1021/jp909004y PMC2892782

[22] Dror RO, Dirks RM, Grossman JP, Xu H, Shaw DE (2012) Biomolecular Simulation: A Computational Microscope for Molecular Biology. Annual Review of Biophysics 41: 429–452. 10.1146/annurev-biophys-042910-155245 22577825

[23] Dixit A, Verkhivker GM (2012) Probing Molecular Mechanisms of the Hsp90 Chaperone: Biophysical Modeling Identifies Key Regulators of Functional Dynamics. PLoS ONE 7: e37605. 22624053 10.1371/journal.pone.0037605 PMC3356286

[24] Papaleo E, Renzetti G, Tiberti M (2012) Mechanisms of Intramolecular Communication in a Hyperthermophilic Acylaminoacyl Peptidase: A Molecular Dynamics Investigation. PLoS ONE 7: e35686. 22558199 10.1371/journal.pone.0035686 PMC3338720

[25] Hayes RL, Noel JK, Mohanty U, Whitford PC, Hennelly SP, et al (2012) Magnesium Fluctuations Modulate RNA Dynamics in the SAM-I Riboswitch. Journal of the American Chemical Society 134: 12043–12053. 22612276 10.1021/ja301454u PMC3675279

[26] Sharma M, Bulusu G, Mitra A (2009) MD simulations of ligand-bound and ligand-free aptamer: Molecular level insights into the binding and switching mechanism of the add A-riboswitch. RNA 15: 1673–1692. 19625387 10.1261/rna.1675809 PMC2743061

[27] Banáš P, Sklenovsky P, Wedekind JE, Sponer J, Otyepka M (2012) Molecular Mechanism of preQ1 Riboswitch Action: A Molecular Dynamics Study. The Journal of Physical Chemistry B 116: 12721–12734. 22998634 10.1021/jp309230v PMC3505677

[28] Doshi U, Kelley JM, Hamelberg D (2012) Atomic-level insights into metabolite recognition and specificity of the SAM-II riboswitch. RNA 18: 300–307. 22194311 10.1261/rna.028779.111 PMC3264916

[29] Priyakumar UD, MacKerell AD Jr (2010) Role of the Adenine Ligand on the Stabilization of the Secondary and Tertiary Interactions in the Adenine Riboswitch. Journal of Molecular Biology 396: 1422–1438. 20026131 10.1016/j.jmb.2009.12.024 PMC2824916

[30] Villa A, Wohnert J, Stock G (2009) Molecular dynamics simulation study of the binding of purine bases to the aptamer domain of the guanine sensing riboswitch. Nucleic Acids Res 37: 4774–4786. 19515936 10.1093/nar/gkp486 PMC2724292

[31] Huang W, Kim J, Jha S, Aboul-ela F (2009) A mechanism for S-adenosyl methionine assisted formation of a riboswitch conformation: A small molecule with a strong arm. Nucleic Acids Research 37: 6528–6539. 19720737 10.1093/nar/gkp664 PMC2770654

[32] Kelley JM, Hamelberg D (2010) Atomistic basis for the on–off signaling mechanism in SAM-II riboswitch. Nucelic Acids Res 38: 1392–1400. 10.1093/nar/gkp1106 PMC3303485 19969538

[33] Petrone PM, Dewhurst J, Tommasi R, Whitehead L, Pomerantz AK (2011) Atomic-scale characterization of conformational changes in the preQ1 riboswitch aptamer upon ligand binding. Journal of Molecular Graphics and Modelling 30: 179–185. 21831681 10.1016/j.jmgm.2011.07.006

[34] Feng J, Walter NG, Brooks CL (2011) Cooperative and Directional Folding of the preQ1 Riboswitch Aptamer Domain. Journal of the American Chemical Society 133: 4196–4199. 21375305 10.1021/ja110411m PMC3109358

[35] Whitford PC, Schug A, Saunders J, Hennelly SP, Onuchic JN, et al (2009) Nonlocal helix formation is key to understanding S-Adenosylmethionine-1 riboswitch function. Biophysical Journal 96: L7–L9. 19167285 10.1016/j.bpj.2008.10.033 PMC2716452

[36] Shaw DE, Deneroff MM, Dror RO, Kuskin JS, Larson RH, et al (2008) Anton, a special-purpose machine for molecular dynamics simulation. Commun ACM 51: 91–97.

[37] Lindorff-Larsen K, Piana S, Dror RO, Shaw DE (2011) How Fast-Folding Proteins Fold. Science 334: 517–520. 22034434 10.1126/science.1208351

[38] Bleckley S, Schroeder SJ (2012) Incorporating global features of RNA motifs in predictions for an ensemble of secondary structures for encapsidated MS2 bacteriophage RNA. RNA 18: 1309–1318. 22645379 10.1261/rna.032326.112 PMC3383962

[39] Kladwang W, Chou F-C, Das R (2012) Automated RNA Structure Prediction Uncovers a Kink-Turn Linker in Double Glycine Riboswitches. Journal of the american chemical society 134: 1404–1407. 22192063 10.1021/ja2093508

[40] Parisien M, Major F (2008) The MC-Fold and MC-Sym pipeline infers RNA structure from sequence data. Nature 452: 51–55. 18322526 10.1038/nature06684

[41] Seetin MG, Mathews DH (2011) Automated RNA tertiary structure prediction from secondary structure and low-resolution restraints. Journal of Computational Chemistry 32: 2232–2244. 21509787 10.1002/jcc.21806 PMC3288334

[42] Weinberg Z, Breaker RR (2011) R2R - software to speed the depiction of aesthetic consensus RNA secondary structures. BMC Bioinformatics 12: 3. 21205310 10.1186/1471-2105-12-3 PMC3023696

[43] Ditzler MA, Otyepka M, Šponer J, Walter NG (2009) Molecular Dynamics and Quantum Mechanics of RNA: Conformational and Chemical Change We Can Believe In. Accounts of Chemical Research 43: 40–47. 10.1021/ar900093g PMC2808146 19754142

[44] Popenda M, Szachniuk M, Antczak M, Purzycka KJ, Lukasiak P, et al (2012) Automated 3D structure composition for large RNAs. Nucleic Acids Research 40: e112. 22539264 10.1093/nar/gks339 PMC3413140

[45] Cruz JA, Blanchet M-F, Boniecki M, Bujnicki JM, Chen S-J, et al (2012) RNA-Puzzles: A CASP-like evaluation of RNA three-dimensional structure prediction. RNA 18: 610–625. 22361291 10.1261/rna.031054.111 PMC3312550

[46] Jossinet F, Ludwig TE, Westhof E (2010) Assemble: an interactive graphical tool to analyze and build RNA architectures at the 2D and 3D levels. Bioinformatics 26: 2057–2059. 20562414 10.1093/bioinformatics/btq321 PMC2916716

[47] Das R, Baker D (2007) Automated de novo prediction of native-like RNA tertiary structures. Proceedings of the National Academy of Sciences 104: 14664–14669. 10.1073/pnas.0703836104 PMC1955458 17726102

[48] Bernauer J, Huang X, Sim AYL, Levitt M (2011) Fully differentiable coarse-grained and all-atom knowledge-based potentials for RNA structure evaluation. RNA 17: 1066–1075. 21521828 10.1261/rna.2543711 PMC3096039

[49] Henin J, Chipot C (2004) Overcoming free energy barriers using unconstrained molecular dynamics simulations. Journal of Chemical Physics 121: 2904–2914. 15291601 10.1063/1.1773132

[50] Hamelberg D, Mongan J, McCammon JA (2004) Accelerated molecular dynamics: A promising and efficient simulation method for biomolecules. The Journal of Chemical Physics 120: 11919–11929. 15268227 10.1063/1.1755656

[51] Sugita Y, Okamoto Y (1999) Replica-exchange molecular dynamics method for protein folding. Chemical Physics Letters 314: 141–151.

[52] Bida JP, Maher LJ (2012) Improved prediction of RNA tertiary structure with insights into native state dynamics. RNA 18: 385–393. 22279150 10.1261/rna.027201.111 PMC3285927

[53] Wenter P, Bodenhausen G, Dittmer J, Pitsch S (2006) Kinetics of RNA Refolding in Dynamic Equilibrium by 1H-Detected 15N Exchange NMR Spectroscopy. Journal of the American Chemical Society 128: 7579–7587. 16756314 10.1021/ja060344a

[54] Hennelly SP, Sanbonmatsu KY (2011) Tertiary contacts control switching of the SAM-I riboswitch. Nucleic Acids Research 39: 2416–2431. 21097777 10.1093/nar/gkq1096 PMC3064774

[55] Hennelly SP, Novikova IV, Sanbonmatsu KY (2013) The expression platform and the aptamer: cooperativity between Mg2+ and ligand in the SAM-I riboswitch. Nucleic Acids Research 41: 1922–1935. 23258703 10.1093/nar/gks978 PMC3562059

[56] Lemieux S, Major F (2006) Automated extraction and classification of RNA tertiary structure cyclic motifs. Nucleic Acids Research 34: 2340–2346. 16679452 10.1093/nar/gkl120 PMC1458283

[57] Boyapati VK, Huang W, Spedale J, Aboul-ela F (2012) Basis for ligand discrimination between ON and OFF state riboswitch conformations: The case of the SAM-I riboswitch. RNA 18: 1230–1243. 22543867 10.1261/rna.032177.111 PMC3358645

[58] Huang N, MacKerell AD (2004) Atomistic view of base flipping in DNA. Philosophical Transactions of the Royal Society of London Series A: Mathematical, Physical and Engineering Sciences 362: 1439–1460. 10.1098/rsta.2004.1383 15306460

[59] Mitton-Fry RM, DeGregorio SJ, Wang J, Steitz TA, Steitz JA (2010) Poly(A) Tail Recognition by a Viral RNA Element Through Assembly of a Triple Helix. Science 330: 1244–1247. 21109672 10.1126/science.1195858 PMC3074936

[60] Lu C, Ding F, Chowdhury A, Pradhan V, Tomsic J, et al (2010) SAM recognition and conformational switching mechanism in the *Bacillus subtilis yitJ* S Box/SAM-I Riboswitch. Journal of Molecular Biology 404: 803–818. 20951706 10.1016/j.jmb.2010.09.059 PMC3222078

[61] Leontis NB, Westhof E (2001) Geometric nomenclature and classification of RNA base pairs. RNA 7: 499–512. 11345429 10.1017/s1355838201002515 PMC1370104

[62] Montange RK, Batey RT (2006) Structure of the S-adenosylmethionine riboswitch regulatory mRNA element. Nature 441: 1172–1175. 16810258 10.1038/nature04819

[63] Montange RK, Mondragón E, van Tyne D, Garst AD, Ceres P, et al (2010) Discrimination between closely related cellular metabolites by the SAM-I riboswitch. Journal of Molecular Biology 396: 761–772. 20006621 10.1016/j.jmb.2009.12.007 PMC2822714

[64] Schroeder Kersten T, Daldrop P, Lilley David MJ (2011) RNA Tertiary Interactions in a Riboswitch Stabilize the Structure of a Kink Turn. Structure 19: 1233–1240. 21893284 10.1016/j.str.2011.07.003 PMC3651934

[65] Heppell B, Blouin S, Dussault A-M, Mulhbacher J, Ennifar E, et al (2011) Molecular insights into the ligand-controlled organization of the SAM-I riboswitch. Nat Chem Biol 7: 384–392. 21532599 10.1038/nchembio.563

[66] Huang W, Kim J, Jha S, Aboul-ela F (2012) Conformational Heterogeneity of the SAM-I Riboswitch Transcriptional ON State: A Chaperone-like Role for S-adenosylmethionine. Journal of molecular biology 418: 331–349. 22425639 10.1016/j.jmb.2012.02.019 PMC4767528

[67] Hayes RL, Noel JK, Mohanty U, Whitford PC, Hennelly SP, et al (2012) Magnesium fluctuations modulate RNA dynamics in the SAM-I riboswitch. Journal of the american chemical society Article ASAP 134: 12043–12053. 10.1021/ja301454u PMC3675279 22612276

[68] Tomsic J, McDaniel BA, Grundy FJ, Henkin TM (2008) Natural variability in S-Adenosylmethionine (SAM)-dependent riboswitches: S-Box elements in *Bacillus subtilis* exhibit differential sensitivity to SAM in vivo and in vitro. Journal of Bacteriology 190: 823–833. 18039762 10.1128/JB.01034-07 PMC2223579

[69] Wickiser JK, Cheah MT, Breaker RR, Crothers DM (2005) The kinetics of ligand binding by an adenine-sensing riboswitch. Biochemistry 44: 13404–13414. 16201765 10.1021/bi051008u

[70] Wickiser JK, Winkler WC, Breaker RR, Crothers DM (2005) The speed of RNA transcription and metabolite binding kinetics operate an FMN riboswitch. Mol Cell 18: 49–60. 15808508 10.1016/j.molcel.2005.02.032

[71] Garst AD, Porter EB, Batey RT (2012) Insights into the Regulatory Landscape of the Lysine Riboswitch. Journal of Molecular Biology 423: 17–33. 22771573 10.1016/j.jmb.2012.06.038 PMC3444622

[72] Serganov A, Patel DJ (2012) Metabolite Recognition Principles and Molecular Mechanisms Underlying Riboswitch Function. Annual Review of Biophysics 41: 343–370. 10.1146/annurev-biophys-101211-113224 PMC4696762 22577823

[73] Montange RK, Batey RT (2008) Riboswitches: emerging themes in RNA structure and function. Annual Review of Biophysics 37: 117–133. 10.1146/annurev.biophys.37.032807.130000 18573075

[74] Zhao P, Zhang W-B, Chen S-J (2010) Predicting Secondary Structural Folding Kinetics for Nucleic Acids. Biophysical Journal 98: 1617–1625. 20409482 10.1016/j.bpj.2009.12.4319 PMC2856163

[75] Greenleaf WJ, Frieda KL, Foster DAN, Woodside MT, Block SM (2008) Direct observation of hierarchical folding in single riboswitch aptamers. Science 319: 630–633. 18174398 10.1126/science.1151298 PMC2640945

[76] Lemay J-F, Penedo JC, Tremblay R, Lilley DMJ, Lafontaine DA (2006) Folding of the Adenine Riboswitch. Chemistry & Biology 13: 857–868. 16931335 10.1016/j.chembiol.2006.06.010

[77] Haller A, Rieder U, Aigner M, Blanchard SC, Micura R (2011) Conformational capture of the SAM-II riboswitch. Nat Chem Biol 7: 393–400. 21532598 10.1038/nchembio.562

[78] Lee M-K, Gal M, Frydman L, Varani G (2010) Real-time multidimensional NMR follows RNA folding with second resolution. Proceedings of the National Academy of Sciences, USA 107: 9192–9197. 10.1073/pnas.1001195107 PMC2889053 20439766

[79] Neupane K, Yu H, Foster DAN, Wang F, Woodside MT (2011) Single-molecule force spectroscopy of the add adenine riboswitch relates folding to regulatory mechanism. Nucleic Acids Research 39: 7677–7687. 21653559 10.1093/nar/gkr305 PMC3177178

[80] Dethoff EA, Petzold K, Chugh J, Casiano-Negroni A, Al-Hashimi HM (2012) Visualizing transient low-populated structures of RNA. Nature 491: 724–728. 23041928 10.1038/nature11498 PMC3590852

[81] Baldwin AJ, Kay LE (2009) NMR spectroscopy brings invisible protein states into focus. Nat Chem Biol 5: 808–814. 19841630 10.1038/nchembio.238

[82] Zhang W, Chen S-J (2006) Exploring the Complex Folding Kinetics of RNA Hairpins: I. General Folding Kinetics Analysis. Biophysical Journal 90: 765–777. 16272440 10.1529/biophysj.105.062935 PMC1367102

[83] Hashem Y, Auffinger P (2009) A short guide for molecular dynamics simulations of RNA systems. Methods 47: 187–197. 18930152 10.1016/j.ymeth.2008.09.020

[84] Banáš P, Hollas D, Zgarbová M, Jurečka P, Orozco M, et al (2010) Performance of Molecular Mechanics Force Fields for RNA Simulations: Stability of UUCG and GNRA Hairpins. Journal of Chemical Theory and Computation 6: 3836–3849. 10.1021/ct100481h PMC8916691 35283696

[85] Andreatta D, Sen S, Perez Lustres JL, Kovalenko SA, Ernsting NP, et al (2006) Ultrafast Dynamics in DNA: “Fraying” at the End of the Helix. Journal of the American Chemical Society 128: 6885–6892. 16719468 10.1021/ja0582105 PMC2528932

[86] Nonin S, Leroy J-L, Gueron M (1995) Terminal Base Pairs of Oligodeoxynucleotides: Imino Proton Exchange and Fraying. Biochemistry 34: 10652–10659. 7654719 10.1021/bi00033a041

[87] Macke Thomas J, Case David A (1997) Modeling Unusual Nucleic Acid Structures. Molecular Modeling of Nucleic Acids: American Chemical Society. pp. 379–393.

[88] Perez A, Marchan I, Svozil D, Šponer J, Cheatham TE, et al (2007) Refinement of the AMBER force field for nucleic acids: improving the description of {alpha}/{gamma} conformers. Biophys J 92: 3817–3829. 17351000 10.1529/biophysj.106.097782 PMC1868997

[89] Onufriev A, Bashford D, Case DA (2000) Modification of the Generalized Born Model Suitable for Macromolecules. The Journal of Physical Chemistry B 104: 3712–3720.

[90] Sim AYL, Levitt M (2011) Clustering to identify RNA conformations constrained by secondary structure. Proceedings of the National Academy of Sciences 108: 3590–3595. 10.1073/pnas.1018653108 PMC3048103 21317361

[91] Giambaşu GM, Lee T-S, Sosa CP, Robertson MP, Scott WG, et al (2010) Identification of dynamical hinge points of the L1 ligase molecular switch. RNA 16: 769–780. 20167653 10.1261/rna.1897810 PMC2844624

