# Supplementary material for: The Impact of a Ligand Binding on Strand Migration in the SAM-I Riboswitch
Source: PLoS Comput Biol. 2013 May 16;9(5):e1003069. doi: 10.1371/journal.pcbi.1003069 (PMC3656099; doi:10.1371/journal.pcbi.1003069)
Supplement: Appendix S1 — Predicting the time scale for the propagation step in strand switching. (DOCX) [file pcbi.1003069.s001.docx]

**Appendix A: Predicting the time scale for the propagation step in strand switching**

Folding rates for large, complex RNAs can be as long as minutes or even days near room temperature, although tRNA folding is thought to take place on a 10-100 microsecond scale. Our simulation assumes that the aptamer core of the riboswitch is already folded. A faster timescale is associated with tRNA [[23](#_ENREF_23),[24](#_ENREF_24)], or with folding of hairpins or short helices [[25](#_ENREF_25)].

A rate constant of 8.6 X 10^4^ s^-1^ for formation of a loop-closing base pair, and 2.3 X 10^-8^ s^-1^ for formation of a base pair extending an already base-paired region have been reported [[26](#_ENREF_26)]. However, it is not clear whether these numbers for base pair formation are valid for a situation in which base pair melting is required for a strand switching.

The paper by Wenter et al[[27](#_ENREF_27)] uses NMR to probe strand switching in an artificial RNA strand switching system, with some resemblance to the SAM-I riboswitch system, in that each duplex contains a two base pair nucleation helix outside of the competition region. Arguing that the data are consistent with a strand switching mechanism in which the rate is limited by complete dissociation of the competing 6 base pair region, as well as the two base pair nucleation region, they observe switching on a timescale of ~0.1-2 s^-1^.

Since the tRNA folding involves the formation of four separate helices, one would expect a significantly slower process than for the three base pair segment modeled here, if helices P1-P3 are already folded. Moreover, binding of SAM produces a non-equilibrium situation which may accelerate strand migration. Therefore we could estimate 10-100 microseconds as an *upper limit* for our expected transition time for the riboswitch strand migration based upon the tRNA folding.

Wenter et al [[27](#_ENREF_27)] vary the temperature in their measurements and extract Arrhenius parameters, which they then argue are comparable to calculated enthalpies for strand dissociation as discussed above. For a single temperature, we estimate free energies at 37 degrees for the starting structure(s) in the simulation, intermediate structures, including the putative rate-limiting transition state, and the final state (Table S1). From the sequence used in the study by Wenter et al, we calculate the predicted free energy of the alternative conformers and the proposed transition states in Table S2. From the two tables, we would estimate the activation Free Energy (as opposed to activation enthalpy) at 14.5 Kcal/mole and 11.5 Kcal/mole for the two conformers in the Wenter et al study, as compared to ~4.9 Kcal/mole for the transition from 2P1_10AT to 6P1_7AT in the SAM-I riboswitch construct. The melting of three base pairs in the SAM-I riboswitch system is therefore predicted to be 6.6-9.6 Kcal/mole more favorable than for the artificial system. Assuming similar values for other parameters for the two systems (for example, the “frequency factor”), we could estimate that the transition would take place 5-7 orders of magnitude faster in the riboswitch system, or ~10^-7^-10^-3^ s (~0.1 microsecond to 1 ms, or perhaps most likely ~1-100 microseconds), if the fully melted state corresponds to the rate-limiting transition state.

A more recent study has used energy landscape analysis of single molecule folding trajectories to derive folding rates for several nucleic acid helices and for an Adenine riboswitch aptamer [[28](#_ENREF_28)]. The reported folding times for all molecules tested were in the range of ~10 μs. An apparent correlation between duplex length and transition time might imply that the transition time could be significantly shorter for the 3 base pair migration simulated in this study, in agreement with our findings. There was little effect of ligand binding observed on the aptamer transition rate, however, though the barrier height was considerably lowered when ligand was added.

To summarize**,** we can expect a strand migration rate of faster than 10-100 microseconds, by analogy with tRNA, assuming that the tRNA folding rate is similar to the unfolding rate, and that the latter is slower than in our system due to the necessity of melting more and longer base-paired regions. By analogy with the system of Wenter et al, we would predict strand migration to occur in the 1-100 microsecond regime, assuming that we can use predicted free energies to estimate activation free energies, and that other parameters associated with the two systems (frequency factor) are of similar order of magnitude. However, the Wenter system estimates rates in an equilibrium system, whereas our system, with SAM added, can be expected to refold more rapidly because it is not at equilibrium.
