# Supplementary material for: The Impact of a Ligand Binding on Strand Migration in the SAM-I Riboswitch
Source: PLoS Comput Biol. 2013 May 16;9(5):e1003069. doi: 10.1371/journal.pcbi.1003069 (PMC3656099; doi:10.1371/journal.pcbi.1003069)
Supplement: Appendix S2 — MC-Sym scripts used to generate starting models for MD simulations. (DOCX) [file pcbi.1003069.s002.docx]

Appendix S2: MC-sym scripts

A. Script for modeling AT helix:

// ==================== Sequence ====================

sequence( r A1 UAAGAAGAGACUUCGGUCUUCUUCUUA )

// (((((((((((....))).))))))))

// AAAAAAAAAAAAAAAAAAAAAAAAAAA

// 123456789012345678901234567

// 1 2

// ==================== Library ====================

//----- Fragment A -----

// stem 1 -> 11, 16 -> 27

ncm_01 = library(

pdb( "MCSYM-DB/2_2/UAUA/*R20*.pdb.gz" )

#1:#2, #3:#4 <- A1:A2, A26:A27

rmsd( 0.1 sidechain && !( pse || lp || hydrogen ) ) )

ncm_02 = library(

pdb( "MCSYM-DB/2_2/AAUU/*R20*.pdb.gz" )

#1:#2, #3:#4 <- A2:A3, A25:A26

rmsd( 0.1 sidechain && !( pse || lp || hydrogen ) ) )

ncm_03 = library(

pdb( "MCSYM-DB/2_2/AGCU/*R20*.pdb.gz" )

#1:#2, #3:#4 <- A3:A4, A24:A25

rmsd( 0.1 sidechain && !( pse || lp || hydrogen ) ) )

ncm_04 = library(

pdb( "MCSYM-DB/2_2/GAUC/*R20*.pdb.gz" )

#1:#2, #3:#4 <- A4:A5, A23:A24

rmsd( 0.1 sidechain && !( pse || lp || hydrogen ) ) )

ncm_05 = library(

pdb( "MCSYM-DB/2_2/AAUU/*R20*.pdb.gz" )

#1:#2, #3:#4 <- A5:A6, A22:A23

rmsd( 0.1 sidechain && !( pse || lp || hydrogen ) ) )

ncm_06 = library(

pdb( "MCSYM-DB/2_2/AGCU/*R20*.pdb.gz" )

#1:#2, #3:#4 <- A6:A7, A21:A22

rmsd( 0.1 sidechain && !( pse || lp || hydrogen ) ) )

ncm_07 = library(

pdb( "MCSYM-DB/2_2/GAUC/*R20*.pdb.gz" )

#1:#2, #3:#4 <- A7:A8, A20:A21

rmsd( 0.1 sidechain && !( pse || lp || hydrogen ) ) )

ncm_08 = library(

pdb( "MCSYM-DB/2_3/AGCUU/*.pdb.gz" )

#1:#2, #3:#5 <- A8:A9, A18:A20

rmsd( 0.1 sidechain && !( pse || lp || hydrogen ) ) )

ncm_09 = library(

pdb( "MCSYM-DB/2_2/GAUC/*R20*.pdb.gz" )

#1:#2, #3:#4 <- A9:A10, A17:A18

rmsd( 0.1 sidechain && !( pse || lp || hydrogen ) ) )

ncm_10 = library(

pdb( "MCSYM-DB/2_2/ACGU/*R20*.pdb.gz" )

#1:#2, #3:#4 <- A10:A11, A16:A17

rmsd( 0.1 sidechain && !( pse || lp || hydrogen ) ) )

ncm_11 = library(

pdb( "MCSYM-DB/6/CUUCGG/*.pdb.gz" )

#1:#6 <- A11:A16

rmsd( 0.5 sidechain && !( pse || lp || hydrogen ) ) )

// ===================== Backtrack =====================

// assemble the whole structure:

structure = backtrack

(

//----- Fragment A -----

// stem 1 -> 11, 16 -> 27

ncm_01

merge( ncm_02 1.5 )

merge( ncm_03 1.5 )

merge( ncm_04 1.5 )

merge( ncm_05 1.5 )

merge( ncm_06 1.5 )

merge( ncm_07 1.5 )

merge( ncm_08 1.5 )

merge( ncm_09 1.5 )

merge( ncm_10 1.5 )

merge( ncm_11 1.5 )

)

// =================== Backtrack Restraints ===================

clash

(

structure

1.5 !( pse || lp || hydrogen )

)

backtrack_rst

(

structure

width_limit = 25%,

height_limit = 33%,

method = probabilistic

)

// =================== Ribose Restraints ===================

ribose_rst

(

structure

method = estimate,

threshold = 2.0

)

// =================== Exploration Initialization =========

explore

(

structure

option(

model_limit = 1000,

time_limit = 30m,

seed = 3210 )

rmsd( 3.0 sidechain && !( pse || lp || hydrogen ) )

pdb( "structure" zipped )

)

B. Script for modeling 6P1_11AT

// ==================== Sequence ====================

sequence( r A1

CUUAUCAAGAGAAGCAGAGGGACUGGCCCGACGAAGCUUCAGCAACCGGUGUAAUGGCGAAAGCCAUG

ACCAAGGUGCUAAAUCCAGCAAGCUCGAACAGCUUGGAAGAUAAGAAGAGACUUCGGUCUUCUUCUUA

)

//

...(((....(((((((((((.[[[[)))...))))))))(((..(((((...(((((....))))).)))..)).)))....]]]]((((((.....))))))...)))((((((

((((....)))).)))))).

// AAA BBBBBBBBBBB CCCCBBB BBBBBBBBDDD DDDDD DDDDD DDDDD DDD

DD DDD CCCCEEEEEE EEEEEE AAFFFFFFFFFFF FFFFFFFFFFF

//

12345678901234567890123456789012345678901234567890123456789012345678901234567

89012345678901234567890123456789012345678901234567890123456

// 1 2 3 4 5 6 7 8 9 10 11

12 13

//

//

//========== Relations ==========

// uncomment the following part for triple helix assumption

implicit_relation(

// these constraints will define the base triple

A1 A113 { pairing }

A2 A112 { pairing }

A3 A111 { pairing }

A4 A110 { pairing }

// A110 A136 { pairing }

// A111 A135 { pairing }

// A112 A134 { pairing }

// A113 A133 { pairing }

)

//relation

//(

// A1:A3 { file( "helixA_RNA" ) stack } 1

//)

// ==================== Library ====================

lnk_30 = library(

pdb( "MCSYM-DB/ss2/CU/*.pdb.gz" )

#1:#2 <- A1:A2

rmsd( 0.1 sidechain && !( pse || lp || hydrogen ) ) )

lnk_20 = library(

pdb( "MCSYM-DB/ss2/UU/*.pdb.gz" )

#1:#2 <- A2:A3

rmsd( 0.1 sidechain && !( pse || lp || hydrogen ) ) )

lnk_10 = library(

pdb( "MCSYM-DB/ss2/UA/*.pdb.gz" )

#1:#2 <- A3:A4

rmsd( 0.1 sidechain && !( pse || lp || hydrogen ) ) )

lnk_00 = library(

pdb( "MCSYM-DB/ss2/AU/*.pdb.gz" )

#1:#2 <- A4:A5

rmsd( 0.1 sidechain && !( pse || lp || hydrogen ) ) )

stem_ABC = library(

pdb( "../3NPB.pdb" )

#8:#112 <- A5:A109

rmsd( 0.1 sidechain && !( pse || lp || hydrogen ) ) )

lnk_01 = library(

pdb( "MCSYM-DB/ss2/AU/*.pdb.gz" )

#1:#2 <- A109:A110

rmsd( 0.1 sidechain && !( pse || lp || hydrogen ) ) )

//lnk_02 = library(

// pdb( "MCSYM-DB/ss2/UA/*.pdb.gz" )

// #1:#2 <- A110:A111

// rmsd( 0.1 sidechain && !( pse || lp || hydrogen ) ) )

stem_D = library(

pdb( "../../yitJ_hybrid/AT/MCsym/*.pdb" )

#1:#27 <- A110:A136

rmsd( 0.1 sidechain && !( pse || lp || hydrogen ) ) )

lnk_03 = library(

pdb( "MCSYM-DB/ss2/UA/*.pdb.gz" )

#1:#2 <- A135:A136

rmsd( 0.1 sidechain && !( pse || lp || hydrogen ) ) )

// ===================== Backtrack =====================

// assemble the whole structure:

structure = backtrack

(

lnk_30

merge( lnk_20 1.5 )

merge( lnk_10 1.5 )

merge( lnk_00 1.5 )

merge( stem_ABC 1.5 )

merge( lnk_01 1.5 )

// merge( lnk_02 1.5 )

merge( stem_D 1.5 )

merge( lnk_03 1.5 )

// ( A3 A2 A1 )

)

// =================== Backtrack Restraints ===================

clash

(

structure

1.5 !( pse || lp || hydrogen )

)

backtrack_rst

(

structure

width_limit = 33%,

height_limit = 33%,

method = probabilistic

)

// =================== Ribose Restraints ===================

ribose_rst

(

structure

method = ccm,

threshold = 1.5

)

// =================== Exploration Initialization =========

explore

(

structure

option(

model_limit = 99999,

seed = 3210 )

rmsd( 3.0 sidechain && !( pse || lp || hydrogen ) )

pdb( "6P1_11AT" zipped )

)
