# Supplementary figures and images for: The Impact of a Ligand Binding on Strand Migration in the SAM-I Riboswitch
Source: PLoS Comput Biol. 2013 May 16;9(5):e1003069. doi: 10.1371/journal.pcbi.1003069 (PMC3656099; doi:10.1371/journal.pcbi.1003069)

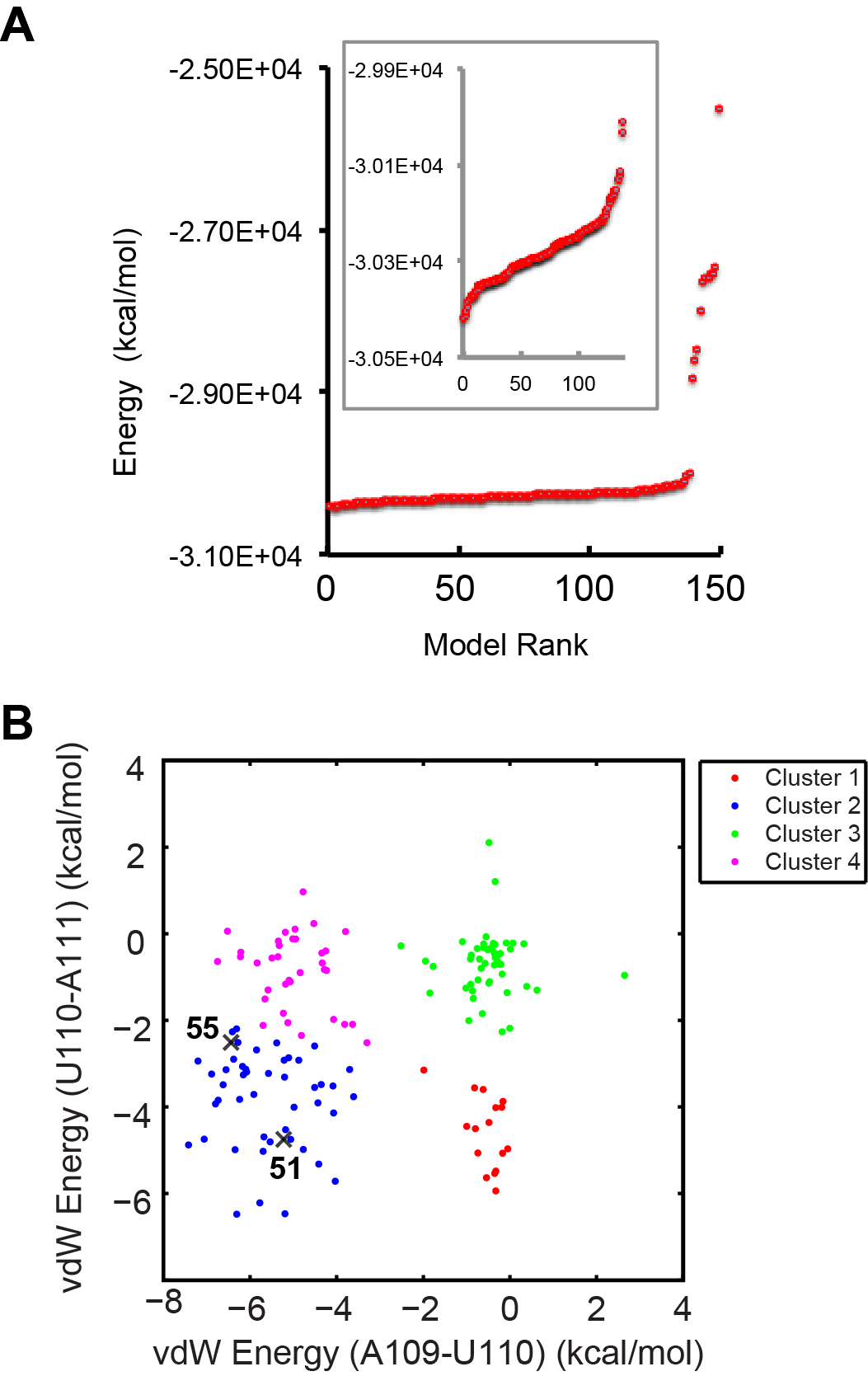

Supplement: Figure S1 — (A) Plot of the free energy rank with amber99 bsc0 and GB solvent model. (B) Scatter plot of vdW_A109-U110 versus vdW_U110-A111 clustered into 4 classes. Model 51 and Model 55 both fall within Cluster 2 (blue) indicating favourable VDW interactions through the A109-A11 junction region. (TIF) [file pcbi.1003069.s003.tif]

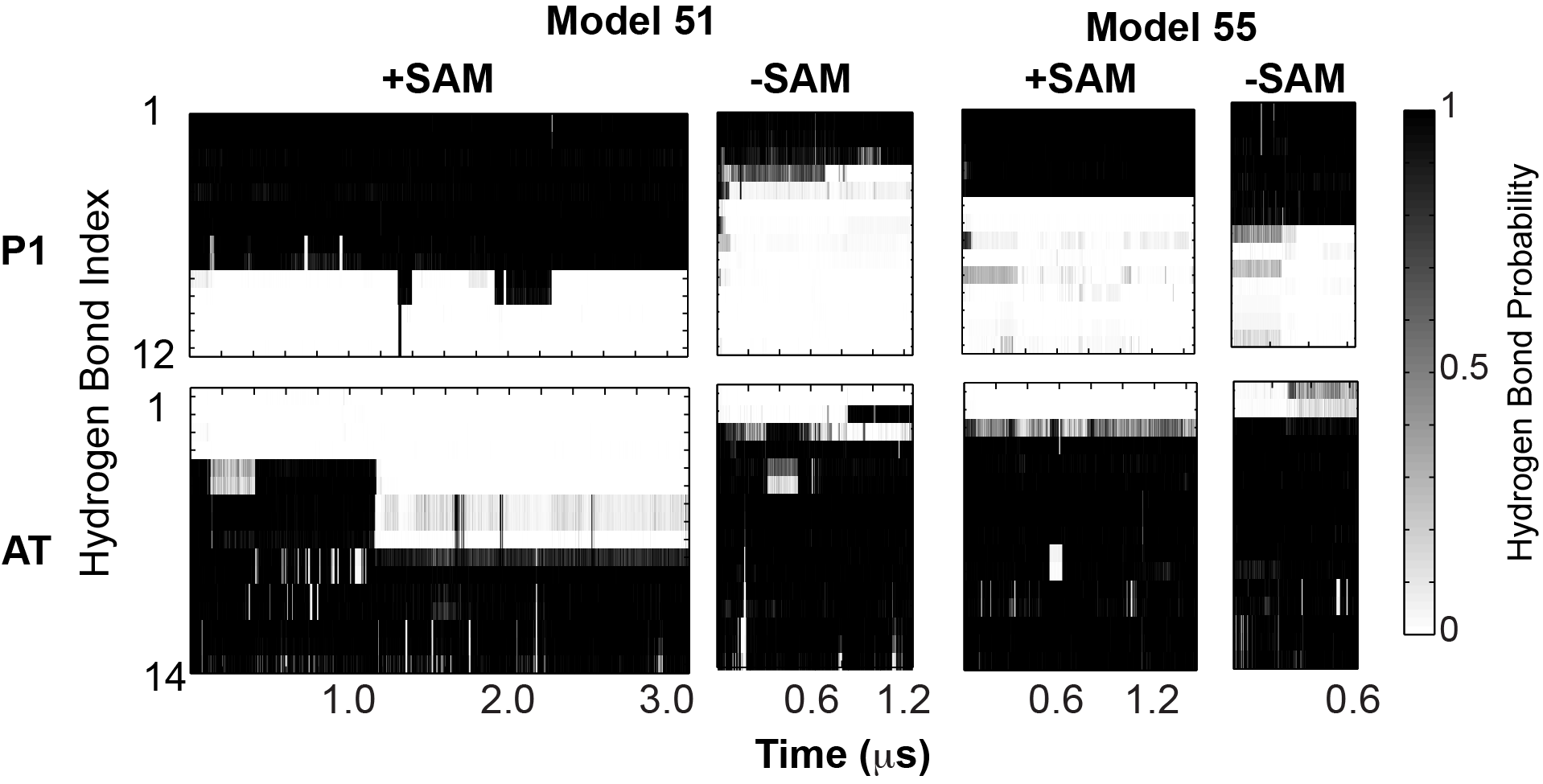

Supplement: Figure S2 — Hydrogen bond analysis of model 51 simulations. Monitor of hydrogen bonds in Watson-Crick base pairs. Here hydrogen bonds are defined with H-bond distance cutoff (<3.5 Å) and H-bond angle cutoff (>145°). The order of hydrogen bonds in the P1 helix is as in Table S3. (TIF) [file pcbi.1003069.s004.tif]

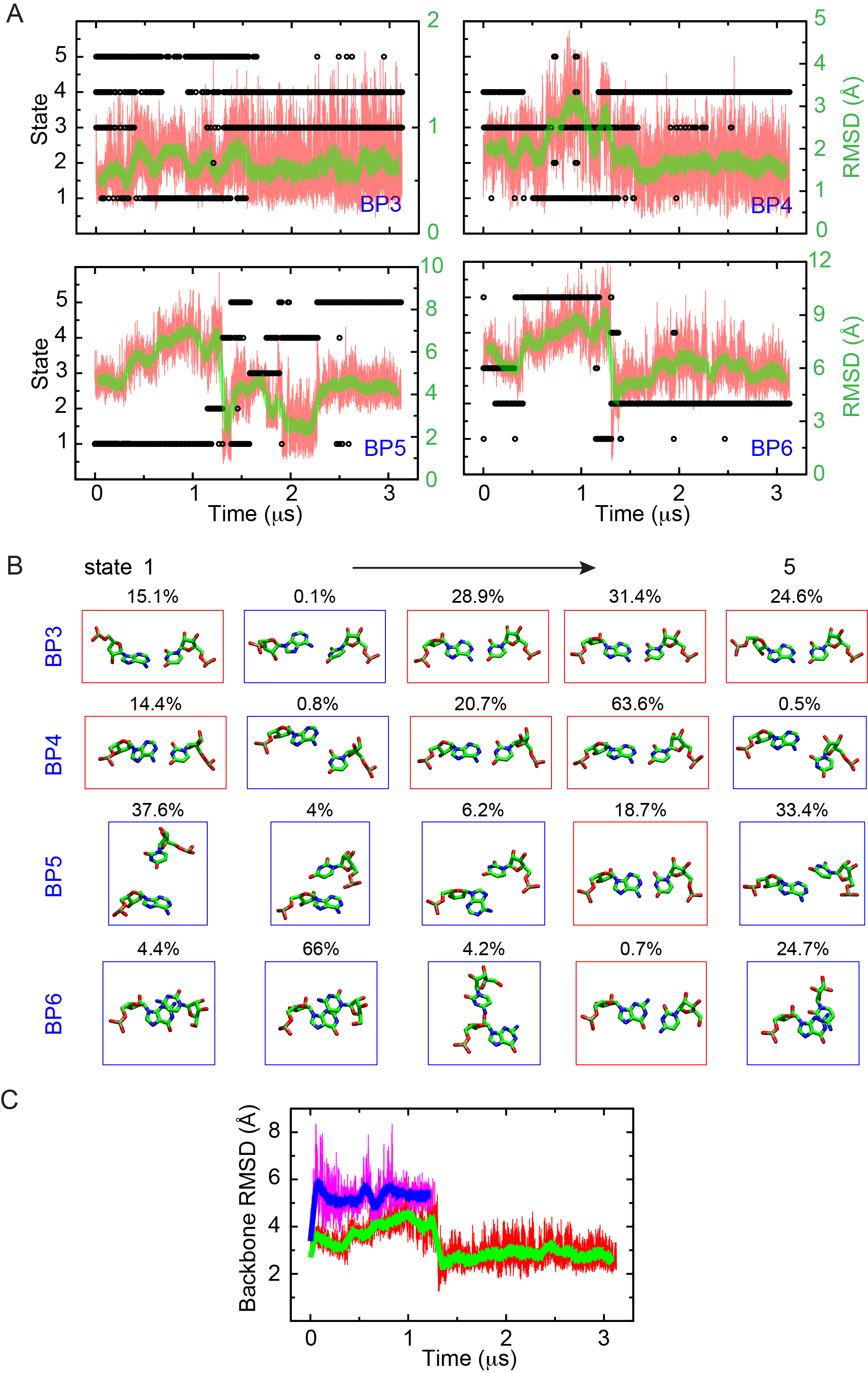

Supplement: Figure S3 — Clustering analysis for base pair 3–6 in the P1 helix for the model 51 trajectory with SAM. Each base pair was grouped into five states using k-means (k = 5) clustering based on the RMSD of nucleobases. (A) State assignments as a function of time for each base pair. The state assigned for each time point is indicated as an open circle. (B) Representative structure for each state. The centroid structure of each state was chosen to represent the state. The population of each state was labeled at the top of individual state. The RMSD for the base pair relative to X-ray coordinates is plotted against the vertical axis on the right. (C) The RMSD for the backbone for P1 helix base pairs 3–6 is plotted along with the same parameter for the trajectory of model 51 without SAM. (TIF) [file pcbi.1003069.s005.tif]

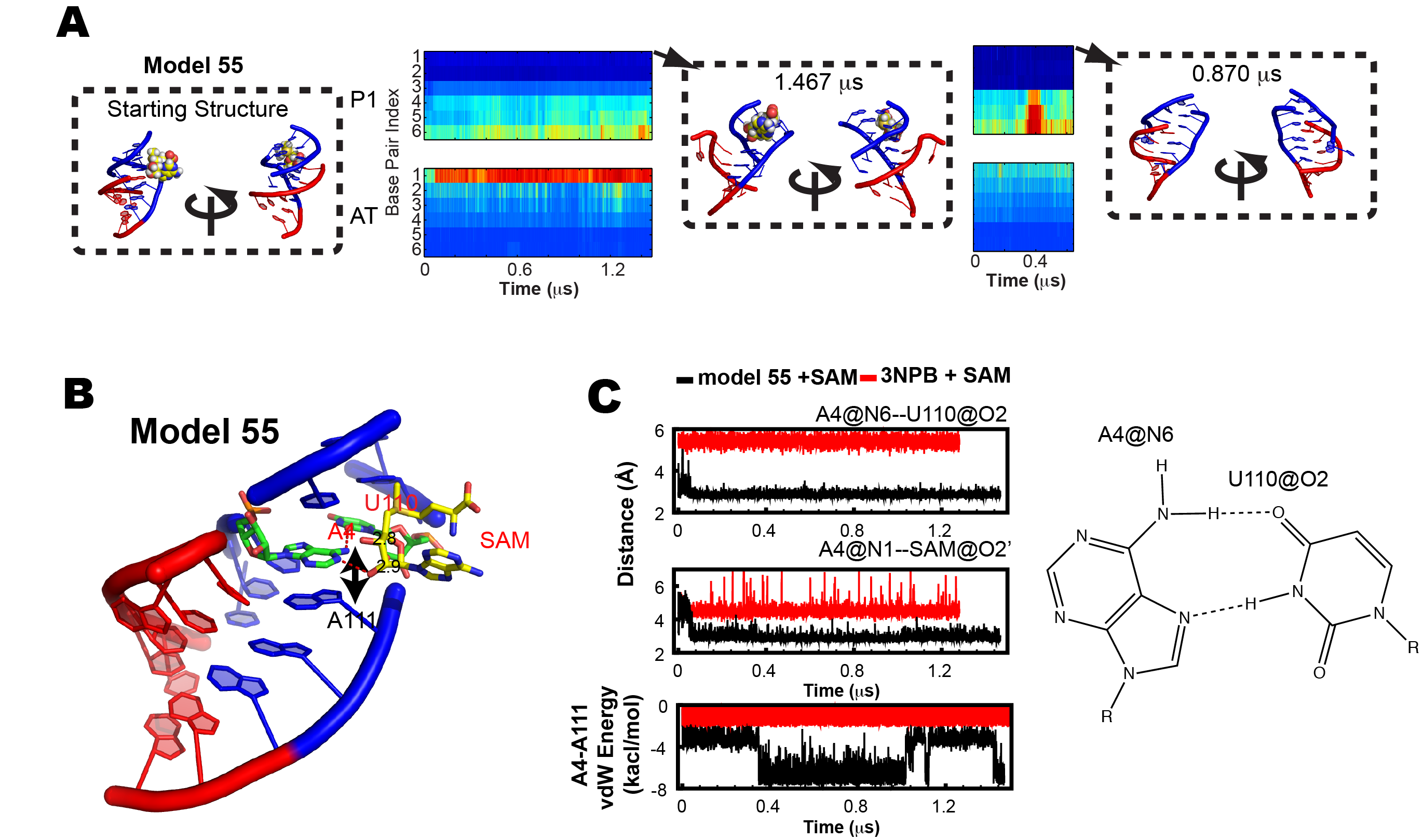

Supplement: Figure S4 — Monitor of some local geometries in model 55 simulations. (A) Time evolution of RMSD for individual base pairs in the P1 and the AT helix from simulations on model 55 in the presence (left) and the absence (right) of SAM, monitored as for model 51 in Figure 3. In contrast to the model 51 trajectories, no migration of P1 or AT helix base pairing is observed beyond the initially unpaired hinge position. (B) Local view of the switching region in model 55. The RNA is shown in cartoon representation except A4 and U100 in stick representation. The ligand SAM is also displayed in stick representation with carbon atoms in yellow. (C) Distance monitor during the simulations for model 55 and 3NPB with SAM for a AU Hoogsteen base pair (Top) and a new interaction between SAM and A4 sampled in model 55 (Middle). Monitor of the base stacking between A4 and A111 via vdW interaction energy (Bottom). The Hoogsteen base pairing scheme is shown on the right. (TIF) [file pcbi.1003069.s006.tif]

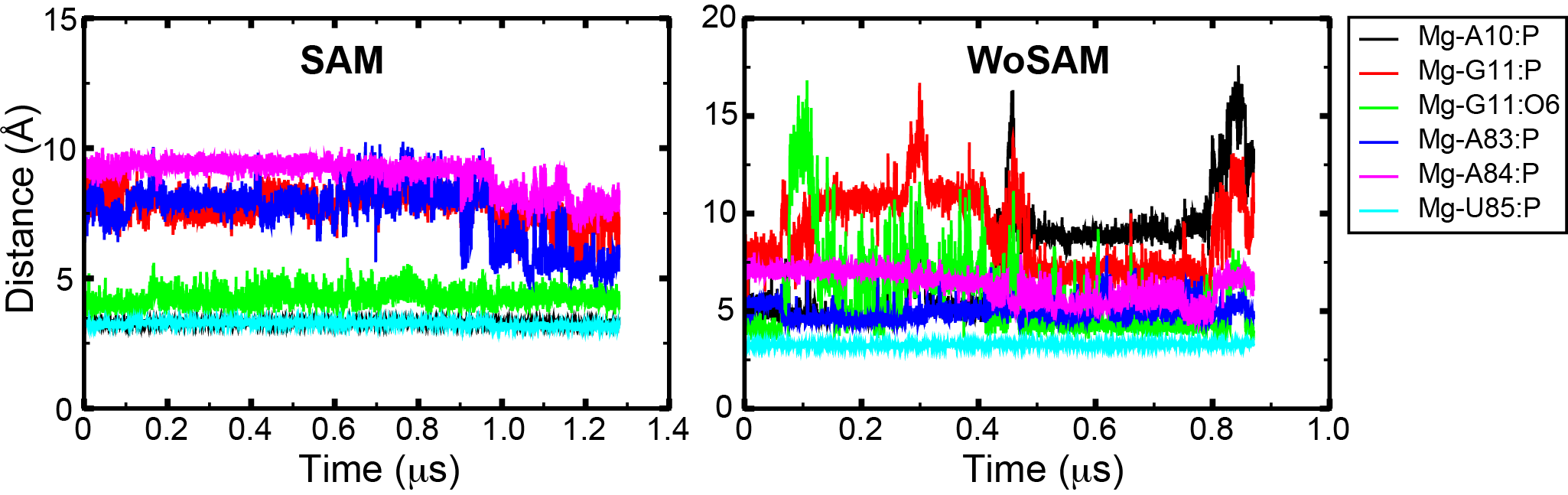

Supplement: Figure S5 — Plots of distance monitor for simulations of the yitJ aptamer (PDB ID: 3NPB) in the presence and the absence of SAM. (TIF) [file pcbi.1003069.s007.tif]

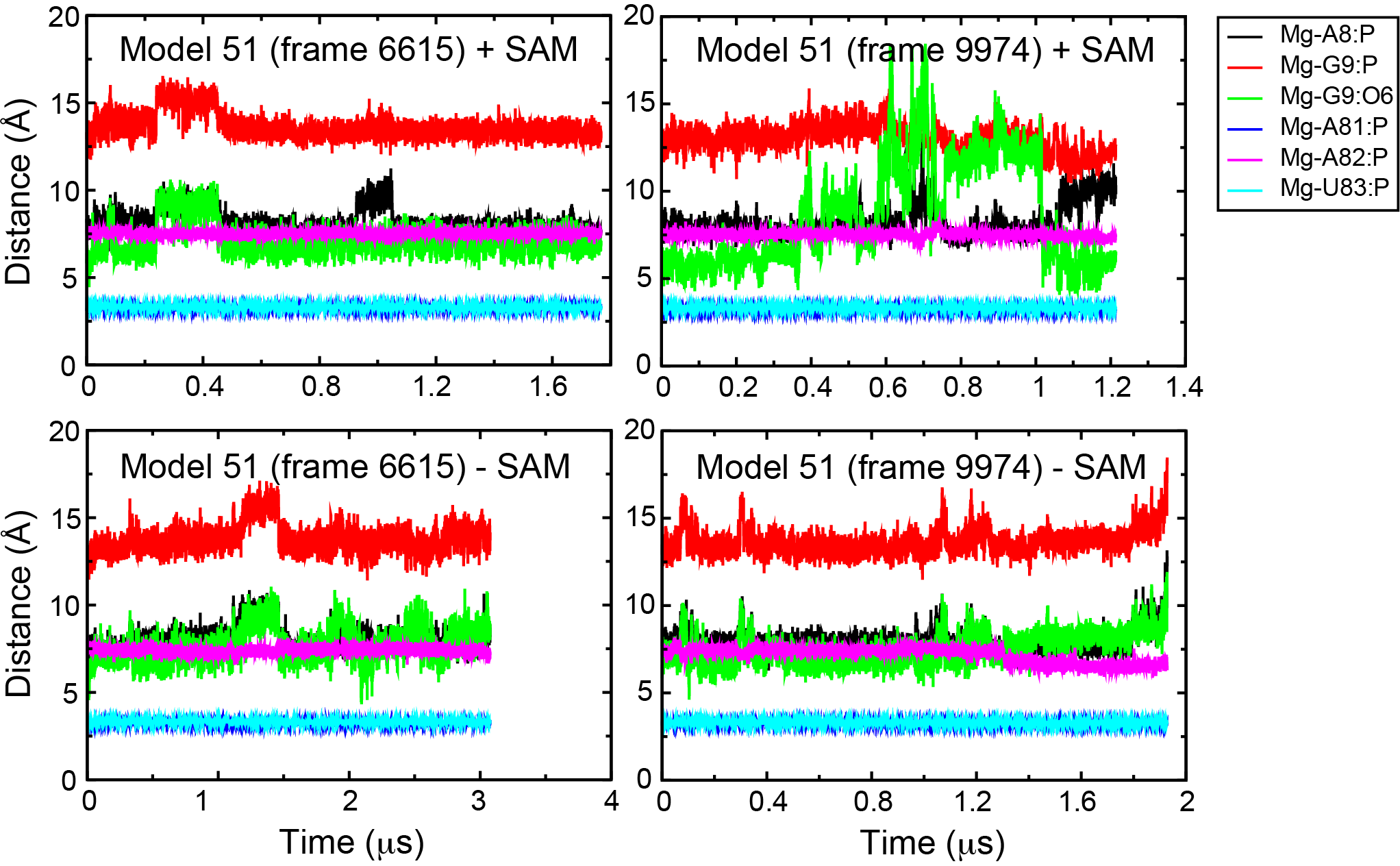

Supplement: Figure S6 — Plots of distance monitor for restarted simulations of model 51 for frame 6615 and frame 9974 in the presence and the absence of SAM. (TIF) [file pcbi.1003069.s008.tif]

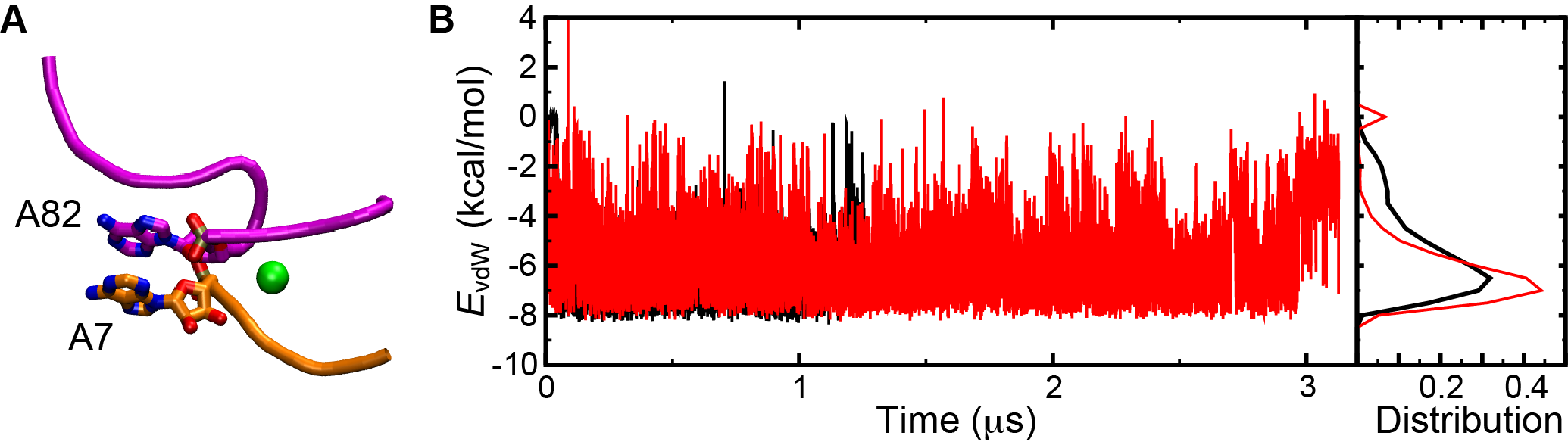

Supplement: Figure S7 — (A) Monitor of vdW stacking energy calculated for non-adjacent dinucleotide stacking between nucleotides 7 and 82 for simulations of model 51 with (red) and without (black) SAM. (B) A histogram of calculated vdW values for the two nucleotides for each simulation. (TIF) [file pcbi.1003069.s009.tif]
