# Supplementary material for: The Impact of a Ligand Binding on Strand Migration in the SAM-I Riboswitch
Source: PLoS Comput Biol. 2013 May 16;9(5):e1003069. doi: 10.1371/journal.pcbi.1003069 (PMC3656099; doi:10.1371/journal.pcbi.1003069)
Supplement: Text S1 — Supplementary Text includes descriptions of: Analysis of MC-sym sampling using the pseudo-dihedral angle, Ranking and selection of MC-sym modeled structures for MD simulations, Supplementary Methods (Details of MD simulations), Captions for supplementary figures and movies. (DOCX) [file pcbi.1003069.s017.docx]

**Analysis of MC-sym sampling using the pseudo-dihedral angle**

A key to bridging the gap between simulation and experimental timescales in this study has been sampling using MC-sym. As a precedent for this approach using proteins, computational techniques were used to model a proposed transition state of Diels-Alder reaction-theozymes were designed and then catalytic sites were engineered into protein scaffolds to de novo generate an artificial enzyme [[1](#_ENREF_1)]. The importance of RNA structure-function relationships has driven the development of theoretical models and implementation of computational tools for RNA tertiary structure modeling [[2-10](#_ENREF_2)]. MC-sym [[5](#_ENREF_5)], has gained popularity due to its established pipeline for structural modeling and the capacity to model relatively large RNA [[11-14](#_ENREF_11)]. We used MC-sym to generate 3D structures for some transition or intermediate state models from secondary structure prediction and SAM binding experiments, while incorporating information from related X-ray coordinates. The importance of RNA structure-function relationships has driven the development of theoretical models and implementation of computational tools for RNA tertiary structure modeling [[2-10](#_ENREF_2)].

**Ranking and selection of MC-sym modeled structures for MD simulations**

Modeled RNA and protein structures are generally ranked according to two types of criteria: agreement with experimental data/constraints and agreement with physical principles guiding biomolecular structure.

In the absence of X-ray coordinates for hybrid SAM-I riboswitch structures we have selected models with coaxial stacking of partial P1 and AT helices. This stacking was demonstrated experimentally by our previous SAM-binding studies[[15](#_ENREF_15)].

To evaluate how well a model complies with fundamental physics for biomolecular folding in solution, it is common to run programs that check compliance with bond length, angle, dihedral constraints and that avoid steric clashes. While these methods can identify unphysical models, they are not useful for ranking amongst those models that pass the quality check. Empirically based potentials, which are sometimes used for protein structures[[16](#_ENREF_16),[17](#_ENREF_17)], are not yet available in widely accepted form for nucleic acids, though empirical databases have informed the original MC-sym modeling.

A more rigorous means of ranking models is to calculate the Free Energy using one of the standard forcefield and parameter sets for nucleic acid and protein modeling and simulations. It follows from the second law of thermodynamics that the probability of formation of an RNA structure at equilibrium and constant temperature will be highest for those structures with the lowest free energy. In the event of a transition between two structures, the Free Energy Landscape will determine the most likely pathway. Our modeling has constrained our starting structure to a specific, non-equilibrium geometry. Statistical mechanics then dictates that of the set of structures satisfying that geometry, those that have the lowest free energy are likely to be sampled for the longest period of time. Moreover, high calculated free energy is an indication that a structure may manifest unstable local structural features, such as steric clashes or electrostatically unfavorable interactions. We therefore decided that it was appropriate to choose the lowest free energy structures as one criterion for the most suitable starting model for the MD simulations.

The energy function we used is composed of Amber 11 force fields for internal energy and the Generalized Born Solvent model for solvation. This kind of physics-based empirical potential has been widely utilized for biological molecular structure prediction and in particular for proteins[[16](#_ENREF_16),[18-20](#_ENREF_18)] for assessing quality of predicted models. It is noted that the use of physics-based potential and statistical approaches such as MM-PB(GB)SA for identifying low energy structures is a logical decision. There is an enormous computational cost for accurate absolute free energy calculations, especially when including explicit solvent. Previous studies demonstrated that both structure and energetics of RNA using a GB model are in agreement with more computationally intense calculations using explicit water or Poisson-Boltzmann continuum methods[[21](#_ENREF_21),[22](#_ENREF_22)]. Since we wished to sample as many structures as possible using MC-sym, free energy calculation incorporating the GB model was chosen as the most efficient metric to rank the pool of models.

**Appendix A: Predicting the time scale for the propagation step in strand switching**

Folding rates for large, complex RNAs can be as long as minutes or even days near room temperature, although tRNA folding is thought to take place on a 10-100 microsecond scale. Our simulation assumes that the aptamer core of the riboswitch is already folded. A faster timescale is associated with tRNA [[23](#_ENREF_23),[24](#_ENREF_24)], or with folding of hairpins or short helices [[25](#_ENREF_25)].

A rate constant of 8.6 X 10^4^ s^-1^ for formation of a loop-closing base pair, and 2.3 X 10^-8^ s^-1^ for formation of a base pair extending an already base-paired region have been reported [[26](#_ENREF_26)]. However, it is not clear whether these numbers for base pair formation are valid for a situation in which base pair melting is required for a strand switching.

The paper by Wenter et al[[27](#_ENREF_27)] uses NMR to probe strand switching in an artificial RNA strand switching system, with some resemblance to the SAM-I riboswitch system, in that each duplex contains a two base pair nucleation helix outside of the competition region. Arguing that the data are consistent with a strand switching mechanism in which the rate is limited by complete dissociation of the competing 6 base pair region, as well as the two base pair nucleation region, they observe switching on a timescale of ~0.1-2 s^-1^.

Since the tRNA folding involves the formation of four separate helices, one would expect a significantly slower process than for the three base pair segment modeled here, if helices P1-P3 are already folded. Moreover, binding of SAM produces a non-equilibrium situation which may accelerate strand migration. Therefore we could estimate 10-100 microseconds as an *upper limit* for our expected transition time for the riboswitch strand migration based upon the tRNA folding.

Wenter et al [[27](#_ENREF_27)] vary the temperature in their measurements and extract Arrhenius parameters, which they then argue are comparable to calculated enthalpies for strand dissociation as discussed above. For a single temperature, we estimate free energies at 37 degrees for the starting structure(s) in the simulation, intermediate structures, including the putative rate-limiting transition state, and the final state (Table S1). From the sequence used in the study by Wenter et al, we calculate the predicted free energy of the alternative conformers and the proposed transition states in Table S2. From the two tables, we would estimate the activation Free Energy (as opposed to activation enthalpy) at 14.5 Kcal/mole and 11.5 Kcal/mole for the two conformers in the Wenter et al study, as compared to ~4.9 Kcal/mole for the transition from 2P1_10AT to 6P1_7AT in the SAM-I riboswitch construct. The melting of three base pairs in the SAM-I riboswitch system is therefore predicted to be 6.6-9.6 Kcal/mole more favorable than for the artificial system. Assuming similar values for other parameters for the two systems (for example, the “frequency factor”), we could estimate that the transition would take place 5-7 orders of magnitude faster in the riboswitch system, or ~10^-7^-10^-3^ s (~0.1 microsecond to 1 ms, or perhaps most likely ~1-100 microseconds), if the fully melted state corresponds to the rate-limiting transition state.

A more recent study has used energy landscape analysis of single molecule folding trajectories to derive folding rates for several nucleic acid helices and for an Adenine riboswitch aptamer [[28](#_ENREF_28)]. The reported folding times for all molecules tested were in the range of ~10 μs. An apparent correlation between duplex length and transition time might imply that the transition time could be significantly shorter for the 3 base pair migration simulated in this study, in agreement with our findings. There was little effect of ligand binding observed on the aptamer transition rate, however, though the barrier height was considerably lowered when ligand was added.

To summarize**,** we can expect a strand migration rate of faster than 10-100 microseconds, by analogy with tRNA, assuming that the tRNA folding rate is similar to the unfolding rate, and that the latter is slower than in our system due to the necessity of melting more and longer base-paired regions. By analogy with the system of Wenter et al, we would predict strand migration to occur in the 1-100 microsecond regime, assuming that we can use predicted free energies to estimate activation free energies, and that other parameters associated with the two systems (frequency factor) are of similar order of magnitude. However, the Wenter system estimates rates in an equilibrium system, whereas our system, with SAM added, can be expected to refold more rapidly because it is not at equilibrium.

**Supplementary Methods**

**Details of MD simulations**

Models satisfying the following criteria were selected for MD simulations: (a) The calculated free energy is favorable using Amber99bsc0 force field with generalized Born (GB) implicit solvent model (Figure S1A); (b) The SAM binding pocket is not occupied; (c) The AT helix and the P1 helix forms coaxial stacking. The GB model igb1 in Amber 11 was used for GB calculations [[22](#_ENREF_22)]. The GB model provides an efficient method for studying energetics and structure of RNA. The SAM ligand was placed in the binding pocket allowing with J1/2 and P3 contacts maintained as observed in the crystal structure. Models showing steric clash with the ligand were removed from the pool. Coaxial stacking was evaluated with the vdW energy for the following two pairs: A109-U110 and U110-A111 (Figure S1B).

For simulations in the presence of SAM, the ligand was placed in the binding pocket with most of the contacts observed in the crystal structure of the aptamer-ligand complex maintained (PDB ID: 3NPB) [[29](#_ENREF_29)]. The only exception were native contacts with A4-U110 in the P1 helix.

These models were well-equilibrated (20 ns) using the program NAMD [[30](#_ENREF_30)] and the Amber99 bsc0 force field for nucleic acids [[31](#_ENREF_31)], ions [[32](#_ENREF_32)] with the TIP3P water model [[33](#_ENREF_33)]. Parameters for SAM were from the Generalized Amber Force Field (GAFF) [[34](#_ENREF_34)] and partial charges were calculated using ANTECHAMBER with the AM1-bcc model [[35-37](#_ENREF_35)]. The starting structures were prepared using the LEaP module in AMBER [[38](#_ENREF_38)]—RNA molecules were solvated in a cubic water box with a 10 Å padding in all directions. 130 sodium ions were placed in the most negatively charged positions around the RNA to neutralize charges of the systems. The systems were energy minimized for 10,000 steps to remove bad contacts. Then, the systems were equilibrated with all heavy atoms restrained harmonically and the temperature raised 10 K per 10,000 steps starting from 0 K to 300 K using temperature reassignment. After reaching the desired temperature, harmonic restrains were gradually reduced using scale from 1.0 to 0 with 0.2 decrement for every 50,000 steps. MD simulations were performed under the NPT ensemble [[39](#_ENREF_39),[40](#_ENREF_40)]. Langevin dynamics was used for constant temperature control, with the value of Langevin coupling coefficient and the Langevin temperature set to 5 ps and 300 K respectively. The pressure was maintained at 1 atm using the Langevin piston method with a period of 100 fs and decay times of 50 fs. A time step of 2 fs was used for all the simulation by using the SHAKE algorithm [[41](#_ENREF_41)] to constrain bonds involving hydrogen atoms.

Well-equilibrated structures after 20 ns of MD simulation were used as inputs for simulations on Anton following the instructions on the Anton wiki (https://wiki.psc.edu/twiki-pub/Anton/). All simulation were also performed following the same MD protocol under the NPT ensemble at 300 K but with a Berendsen thermostat and barostat [[42](#_ENREF_42)]. Snapshots were save for every 200 ps. A summary of all the simulations are provided in Table 1.

Appendix B: Scripts for Generating Nucleic Acid Models Using MC-sym

**SI Figures**

**Figure S1.** (A) Plot of the free energy rank with amber99 bsc0 and GB solvent model. (B) Scatter plot of vdW_A109-U110 versus vdW_U110-A111 clustered into 4 classes. Model 51 and Model 55 both fall within Cluster 2 (blue) indicating favourable VDW interactions through the A109-A11 junction region.

**Figure S2. Hydrogen bond analysis of model 51 simulations.** Monitor of hydrogen bonds in Watson-Crick base pairs. Here hydrogen bonds are defined with H-bond distance cutoff (< 3.5 Å) and H-bond angle cutoff (> 145**°**). The order of hydrogen bonds in the P1 helix is as in Table S2.

**Figure S3. Clustering analysis for base pair 3-6 in the P1 helix for the model 51 trajectory with SAM**. Each base pair was grouped into five states using *k*-means (*k* = 5) clustering based on the RMSD of nucleobases. (A) State assignments as a function of time for each base pair. The state assigned for each time point is indicated as an open circle. (B) Representative structure for each state. The centroid structure of each state was chosen to represent the state. The population of each state was labeled at the top of individual state. The RMSD for the base pair relative to X-ray coordinates is plotted against the vertical axis on the right. (C) The RMSD for the backbone for P1 helix base pairs 3-6 is plotted along with the same parameter for the trajectory of model 51 without SAM.

**Figure S4. Monitor of some local geometries in model 55 simulations.** (A) Time evolution of RMSD for individual base pairs in the P1 and the AT helix from simulations on model 55 in the presence (left) and the absence (right) of SAM, monitored as for model 51 in Figure 3. In contrast to the model 51 trajectories, no migration of P1 or AT helix base pairing is observed beyond the initially unpaired hinge position. (B) Local view of the switching region in model 55. The RNA is shown in cartoon representation except A4 and U100 in stick representation. The ligand SAM is also displayed in stick representation with carbon atoms in yellow. (C) Distance monitor during the simulations for model 55 and 3NPB with SAM for a AU Hoogsteen base pair (Top) and a new interaction between SAM and A4 sampled in model 55 (Middle). Monitor of the base stacking between A4 and A111 via vdW interaction energy (Bottom). The Hoogsteen base pairing scheme is shown on the right.

**Figure S5**. **Mg^2+^ contact distances in *yitJ* aptamer simulations**. Plots of distance monitor for simulations of the *yitJ* aptamer (PDB ID: 3NPB) in the presence and the absence of SAM.

**Figure S6**. **Mg^2+^ contact distances for restarted simulations for model 51.** Plots of distance monitor for restarted simulations of frame 6615 and frame 9974 from model 51 with SAM simulation in the presence and the absence of SAM.

**Figure S7. Non-adjacent dinucleotide stacking between J1/2 and J3/4.** (A) Monitor of vDW stacking energy calculated for non-adjacent dinucleotide stacking between residues 7 and 82 for simulations of model 51 with (red) and without (black) SAM. (B) A histogram of calculated vDW values for the two residues for each simulation.

**Supplementary Movies**

**Movie S1.** Visualization of trajectory for model 51, with SAM, highlighting strand migration event converting 3 base pairs from AT to P1 helix configuration.

**Movie S2.** Visualization of SAM, core Mg^2+^, electronegative moieties in J1/2 and J3/4, and the dinucleotide stack between residues A7/9 in J1/2 and A82/84 in J3/4. The bases in the non-adjacent dinucleotide stack are highlighted as sticks. G9/11 O6 is highlighted as a purple or red sphere. The Mg^2+^ ion is depicted as a yellow sphere. Backbone phosphates are also highlighted.

**Movie S3.** Similar visualization as for S2 for model 51 without SAM

**Movie S4 and S5.** Similar visualizations for trajectories involving model 55 with (**S4)** and without (**S5**) SAM.

**Movie S6 and S7.** Similar visualizations for trajectories involving the aptamer, starting from x-ray coordinates, with (**S6**) without (**S7**) SAM.

**References**

1. Siegel JB, Zanghellini A, Lovick HM, Kiss G, Lambert AR, et al. (2010) Computational Design of an Enzyme Catalyst for a Stereoselective Bimolecular Diels-Alder Reaction. Science 329: 309-313.

2. Jossinet F, Ludwig TE, Westhof E (2010) Assemble: an interactive graphical tool to analyze and build RNA architectures at the 2D and 3D levels. Bioinformatics 26: 2057-2059.

3. Das R, Baker D (2007) Automated de novo prediction of native-like RNA tertiary structures. Proceedings of the National Academy of Sciences 104: 14664-14669.

4. Kladwang W, Chou F-C, Das R (2011) Automated RNA structure prediction uncovers a missing link in double glycine riboswitches. Quantitative Biology. pp. 1-21.

5. Parisien M, Major F (2008) The MC-Fold and MC-Sym pipeline infers RNA structure from sequence data. Nature 452: 51-55.

6. Jonikas MA, Radmer RJ, Laederach A, Das R, Pearlman S, et al. (2009) Coarse-grained modeling of large RNA molecules with knowledge-based potentials and structural filters. RNA 15: 189-199.

7. Sharma S, Ding F, Dokholyan NV (2008) iFoldRNA: three-dimensional RNA structure prediction and folding. Bioinformatics 24: 1951-1952.

8. Bernauer J, Huang X, Sim AYL, Levitt M (2011) Fully differentiable coarse-grained and all-atom knowledge-based potentials for RNA structure evaluation. RNA.

9. Pasquali S, Derreumaux P (2010) HiRE-RNA: A High Resolution Coarse-Grained Energy Model for RNA. The Journal of Physical Chemistry B 114: 11957-11966.

10. Paliy M, Melnik R, Shapiro BA (2010) Coarse-graining RNA nanostructures for molecular dynamics simulations. Physical Biology 7: 036001.

11. Yang H, Wang Z, Shen Y, Wang P, Jia X, et al. (2010) Crystal Structure of the Nosiheptide-Resistance Methyltransferase of Streptomyces actuosus. Biochemistry 49: 6440-6450.

12. Ali M, Lipfert J, Seifert S, Herschlag D, Doniach S (2010) The ligand-free state of the TPP riboswitch: A partially folded RNA structure. Journal of Molecular Biology 396: 153-165.

13. Reymond C, Lévesque D, Bisaillon M, Perreault J-P (2010) Developing Three-Dimensional Models of Putative-Folding Intermediates of the HDV Ribozyme. Structure (London, England : 1993) 18: 1608-1616.

14. Sim AYL, Levitt M (2011) Clustering to identify RNA conformations constrained by secondary structure. Proceedings of the National Academy of Sciences 108: 3590-3595.

15. Boyapati VK, Huang W, Spedale J, Aboul-ela F (2012) Basis for ligand discrimination between ON and OFF state riboswitch conformations: The case of the SAM-I riboswitch. RNA 18: 1230-1243.

16. Kihara D, Chen H, Yang YD (2009) Quality Assessment of Protein Structure Models. Current Protein & Peptide Science 10: 216-228.

17. Narang P, Bhushan K, Bose S, Jayaram B (2006) Protein Structure Evaluation using an All-Atom Energy Based Empirical Scoring Function. Journal of Biomolecular Structure and Dynamics 23: 385-406.

18. Lee MC, Duan Y (2004) Distinguish protein decoys by Using a scoring function based on a new AMBER force field, short molecular dynamics simulations, and the generalized born solvent model. Proteins: Structure, Function, and Bioinformatics 55: 620-634.

19. Wroblewska L, Skolnick J (2007) Can a physics-based, all-atom potential find a protein's native structure among misfolded structures? I. Large scale AMBER benchmarking. Journal of Computational Chemistry 28: 2059-2066.

20. Laing C, Schlick T (2010) Computational approaches to 3D modeling of RNA Journal of Physics: Condensed Matter 22: 283101/283101-283101/283118.

21. Tsui V, Case DA (2000) Molecular Dynamics Simulations of Nucleic Acids with a Generalized Born Solvation Model. Journal of the American Chemical Society 122: 2489-2498.

22. Tsui V, Case DA (2000) Theory and applications of the generalized Born solvation model in macromolecular simulations. Biopolymers 56: 275-291.

23. Thirumalai D, Woodson SA (1996) Kinetics of Folding of Proteins and RNA. Accounts of Chemical Research 29: 433-439.

24. Bina-Stein M, Crothers DM (1975) Localization of the structural change induced in tRNA fMet (Escherichia coli) by acidic pH. Biochemistry 14: 4185-4191.

25. Cisse II, Kim H, Ha T (2012) A rule of seven in Watson-Crick base-pairing of mismatched sequences. Nat Struct Mol Biol 19: 623-627.

26. Zhang W, Chen S-J (2006) Exploring the Complex Folding Kinetics of RNA Hairpins: I. General Folding Kinetics Analysis. Biophysical Journal 90: 765-777.

27. Wenter P, Bodenhausen G, Dittmer J, Pitsch S (2006) Kinetics of RNA Refolding in Dynamic Equilibrium by 1H-Detected 15N Exchange NMR Spectroscopy. Journal of the American Chemical Society 128: 7579-7587.

28. Neupane K, Ritchie DB, Yu H, Foster DAN, Wang F, et al. (2012) Transition Path Times for Nucleic Acid Folding Determined from Energy-Landscape Analysis of Single-Molecule Trajectories. Physical Review Letters 109: 068102.

29. Lu C, Ding F, Chowdhury A, Pradhan V, Tomsic J, et al. (2010) SAM recognition and conformational switching mechanism in the *Bacillus subtilis yitJ* S Box/SAM-I Riboswitch. Journal of Molecular Biology 404: 803-818.

30. Phillips JC, Braun R, Wang W, Gumbart J, Tajkhorshid E, et al. (2005) Scalable molecular dynamics with NAMD. J Comput Chem 26: 1781-1802.

31. Perez A, Marchan I, Svozil D, Sponer J, Cheatham TE, 3rd, et al. (2007) Refinement of the AMBER force field for nucleic acids: improving the description of alpha/gamma conformers. Biophys J 92: 3817-3829.

32. Aqvist J (1990) Ion Water Interaction Potentials Derived from Free-Energy Perturbation Simulations. Journal of Physical Chemistry 94: 8021-8024.

33. Jorgensen WL, Chandrasekhar J, Madura JD, Impey RW, Klein ML (1983) Comparison of Simple Potential Functions for Simulating Liquid Water. Journal of Chemical Physics 79: 926-935.

34. Wang J, Wolf RM, Caldwell JW, Kollman PA, Case DA (2004) Development and testing of a general amber force field. J Comput Chem 25: 1157-1174.

35. Wang J, Wang W, Kollman PA, Case DA (2006) Automatic atom type and bond type perception in molecular mechanical calculations. J Mol Graph Model 25: 247-260.

36. Jakalian A, Jack DB, Bayly CI (2002) Fast, efficient generation of high-quality atomic charges. AM1-BCC model: II. Parameterization and validation. Journal of Computational Chemistry 23: 1623-1641.

37. Jakalian A, Bush BL, Jack DB, Bayly CI (2000) Fast, efficient generation of high-quality atomic Charges. AM1-BCC model: I. Method. Journal of Computational Chemistry 21: 132-146.

38. Case DA, Cheatham TE, 3rd, Darden T, Gohlke H, Luo R, et al. (2005) The Amber biomolecular simulation programs. J Comput Chem 26: 1668-1688.

39. Martyna GJ, Tobias DJ, Klein ML (1994) Constant-Pressure Molecular-Dynamics Algorithms. Journal of Chemical Physics 101: 4177-4189.

40. Feller SE, Zhang YH, Pastor RW, Brooks BR (1995) Constant-Pressure Molecular-Dynamics Simulation - the Langevin Piston Method. Journal of Chemical Physics 103: 4613-4621.

41. Ryckaert J-P, Ciccotti G, Berendsen HJC (1977) Numerical integration of the cartesian equations of motion of a system with constraints: molecular dynamics of n-alkanes. Journal of Computational Physics 23: 327-341.

42. Berendsen HJC, Postma JPM, Vangunsteren WF, Dinola A, Haak JR (1984) Molecular-Dynamics with Coupling to an External Bath. Journal of Chemical Physics 81: 3684-3690.
