# Supplementary material for: The Impact of a Ligand Binding on Strand Migration in the SAM-I Riboswitch
Source: PLoS Comput Biol. 2013 May 16;9(5):e1003069. doi: 10.1371/journal.pcbi.1003069 (PMC3656099; doi:10.1371/journal.pcbi.1003069)
Supplement: Table S1 — Calculated Free Energies (kcal/mol) using RNAeval (30) for formation of variant secondary structures of the riboswitch conformation shown in Figure 1 (DOCX) [file pcbi.1003069.s018.docx]

**Table S1.** Calculated Free Energies (kcal/mol) using RNAeval ([30](#_ENREF_30)) for formation of variant secondary structures of the riboswitch conformation shown in Figure 1

| **Construct Name** | **Energy (kcal/mol)** |
| --- | --- |
| 2P1_10AT | -42.10 |
| 3P1_10AT | -42.50 |
| 3P1_7AT | -37.60 |
| 4P1_9AT | -42.30 |
| 5P1_6AT | -38.8 |
| 5P1_7AT | -39.4 |
| 5P1_8AT | -42.6 |
| 6P1_6AT | -41.3 |
| 6P1_7AT | -41.1 |
| 8P1_5AT | -42.5 |
| 2P1_5AT | -34.9 |
| 3P1_5AT | -36.0 |

.
