# Supplementary material for: The Impact of a Ligand Binding on Strand Migration in the SAM-I Riboswitch
Source: PLoS Comput Biol. 2013 May 16;9(5):e1003069. doi: 10.1371/journal.pcbi.1003069 (PMC3656099; doi:10.1371/journal.pcbi.1003069)
Supplement: Table S2 — Calculated Free Energies using RNAeval for putative secondary structures and transition states from the study by Wenter et al. 2006. (DOCX) [file pcbi.1003069.s019.docx]

**Table S2**. Calculated Free Energies using RNAeval for putative secondary structures and transition states from the study by Wenter et al. 2006.

| **State** | **Energy (kcal/mol)** |
| --- | --- |
| Fold A | -14.9 |
| Fold B | -11.9 |
| Unfold A | -0.4 |
| Unfold B | -0.4 |
