# Supplementary material for: The Impact of a Ligand Binding on Strand Migration in the SAM-I Riboswitch
Source: PLoS Comput Biol. 2013 May 16;9(5):e1003069. doi: 10.1371/journal.pcbi.1003069 (PMC3656099; doi:10.1371/journal.pcbi.1003069)
Supplement: Table S3 — List of hydrogen bonds included in calculations shown in Figures 5&6 and Figure S2. (DOCX) [file pcbi.1003069.s020.docx]

**Table S3.** List of hydrogen bonds included in calculations shown in Figures 5&6 and Figure S2.

| **Helix** | **Index** | **HBD** | **H** | **HBA** |
| --- | --- | --- | --- | --- |
| **P1** | 1 | 108@N2 | 108@H21 | 6@O2 |
|  | 2 | 108@N1 | 108@H1 | 6@N3 |
|  | 3 | 6@N4 | 6@H41 | 108@O6 |
|  | 4 | 5@N3 | 5@H3 | 109@N1 |
|  | 5 | 109@N6 | 109@H61 | 5@O4 |
|  | 6 | 4@N6 | 4@H61 | 110@O4 |
|  | 7 | 110@N3 | 110@H3 | 4@N1 |
|  | 8 | 3@N3 | 3@H3 | 111@N1 |
|  | 9 | 111@N6 | 111@H61 | 3@O4 |
|  | 10 | 2@N3 | 2@H3 | 112@N1 |
|  | 11 | 112@N6 | 112@H61 | 2@O4 |
|  | 12 | 113@N2 | 113@H21 | 1@O2 |
|  | 13 | 113@N1 | 113@H1 | 1@N3 |
|  | 14 | 1@N4 | 1@H41 | 113@O6 |
| **AT** | 1 | 136@N6 | 136@H61 | 110@O4 |
|  | 2 | 110@N3 | 110@H3 | 136@N1 |
|  | 3 | 135@N3 | 135@H3 | 111@N1 |
|  | 4 | 111@N6 | 111@H61 | 135@O4 |
|  | 5 | 134@N3 | 134@H3 | 112@N1 |
|  | 6 | 112@N6 | 112@H61 | 134@O4 |
|  | 7 | 113@N2 | 113@H21 | 133@O2 |
|  | 8 | 113@N1 | 113@H1 | 133@N3 |
|  | 9 | 133@N4 | 133@H41 | 113@O6 |
|  | 10 | 132@N3 | 132@H3 | 114@N1 |
|  | 11 | 114@N6 | 114@H61 | 132@O4 |
|  | 12 | 131@N3 | 131@H3 | 115@N1 |
|  | 13 | 115@N6 | 115@H61 | 131@O4 |
|  | 14 | 116@N2 | 116@H21 | 130@O2 |
|  | 15 | 116@N1 | 116@H1 | 130@N3 |
|  | 16 | 130@N4 | 130@H41 | 116@O6 |
